# Metabolic control of methylation landscapes in cancer growth and plasticity: targeting the epigenetic–metabolic axis with natural compounds and emerging therapies

**DOI:** 10.3389/fcell.2026.1943655

**Published:** 2026-10-14

**Authors:** Qi Liu, Ying Yu, Wenbo Li, Xinxu Chen, Zekai Shi, Gang Li, Shibo Wei, Zongjian Luo

**Affiliations:** 1 Changchun University of Chinese Medicine, Changchun, China; 2 Affiliated Hospital of Changchun University of Chinese Medicine, Changchun, China; 3 School of Basic Medicine, Faculty of Medicine, Dalian University of Technology, Dalian, China; 4 China Academy of Chinese Medical Sciences Guang’anmen Hospital, Beijing, China

**Keywords:** cancer cell plasticity, epigenetic reprogramming, methylation, S-adenosylmethionine, therapy resistance

## Abstract

Cancer cell plasticity enables tumor cells to dynamically transition between diverse phenotypic states, including epithelial–mesenchymal plasticity stemness, dormancy, lineage switching, immune evasion, and therapy-resistant phenotypes. Although metabolic reprogramming and epigenetic regulation have each been recognized as hallmarks of cancer, the mechanistic framework linking metabolic adaptation to dynamic cell-state transitions remains incompletely understood. In this Review, we propose that methylation serves as a major mechanistic interface through which metabolic rewiring can influence adaptive cancer cell plasticity. We discuss how one-carbon metabolism regulates the availability of the universal methyl donor S-adenosylmethionine (SAM), thereby influencing DNA methylation, histone methylation, and multiple forms of RNA methylation, with N6-methyladenosine (m^6^A) representing the principal RNA modification discussed in this review. Rather than responding uniformly to metabolic change, these methylation systems represent distinct but metabolically connected regulatory layers that share dependence on one-carbon metabolism while differing in their biochemical sensitivities and regulatory outputs. Net methylation states, however, are also shaped by metabolically constrained demethylation, including α-ketoglutarate-, oxygen-, Fe^2+^-, and flavin adenine dinucleotide (FAD)-dependent eraser pathways. Together, these metabolically responsive writing and erasing processes influence transcriptional stability, chromatin accessibility, and post-transcriptional regulation during reversible cell-state transitions. We further examine how environmental and therapeutic stresses, including hypoxia, nutrient limitation, immune surveillance, and anticancer therapy, reshape methylation programs through metabolic adaptation, and how such remodeling may support phenotypic flexibility and survival under selective pressure. Building upon these advances, we propose the concept of the adaptive methylation landscape, a perturbation-responsive, state-associated, and potentially reversible distribution of methylation states that contributes to shaping the spectrum of cellular states accessible during tumor evolution. Finally, we discuss emerging therapeutic strategies targeting the metabolic-methylation axis and highlight how single-cell multi-omics, spatial epigenomics, functional epigenome editing, and AI may enable dynamic mapping of adaptive methylation landscapes. We propose that cancer cell plasticity is not merely a genetic phenomenon but can also be shaped by metabolically responsive methylation programs, providing a testable conceptual framework for future precision epigenetic therapies.

## 1 Introduction

Cancer progression is increasingly recognized as a dynamic evolutionary process driven not only by genetic alterations but also by the remarkable ability of tumor cells to adapt to changing environmental and therapeutic pressures. This adaptive capacity, commonly referred to as cancer cell plasticity, enables malignant cells to reversibly transition between distinct cellular states, including EMP, encompassing epithelial-mesenchymal transition (EMT) and hybrid epithelial–mesenchymal states, stem-like reprogramming, dormancy, lineage switching, and therapy-resistant phenotypes (Quintanal-Villalonga et al., 2020; Perez-Gonzalez et al., 2023). Such plasticity is now considered a fundamental determinant of tumor progression, metastatic dissemination, immune evasion, and treatment failure.

Historically, cancer metabolism, epigenetic regulation, and cellular plasticity have largely been investigated as distinct yet interconnected fields of cancer research (Hanahan, 2022). Metabolic reprogramming has been viewed primarily as a mechanism that fulfills the energetic and biosynthetic demands of rapidly proliferating cancer cells. In parallel, epigenetic alterations, particularly DNA methylation and histone modifications, have been studied as regulatory mechanisms governing gene expression and chromatin architecture (Vander Heiden et al., 2009). More recently, RNA methylation has emerged as an additional layer of post-transcriptional regulation that contributes to tumor initiation and progression (He et al., 2019). Meanwhile, cancer cell plasticity has been extensively investigated as a phenotypic consequence of oncogenic signaling and microenvironmental adaptation (Perez-Gonzalez et al., 2023). Despite substantial progress in each of these fields, the mechanistic links connecting metabolic rewiring to cell-state transitions remain incompletely understood.

Accumulating evidence suggests that methylation may serve as a major mechanistic interface linking metabolic adaptation to cancer cell plasticity (Lu and Thompson, 2012; Atlasi and Stunnenberg, 2017). Among epigenetic modifications, methylation is particularly sensitive to metabolic state because methyl-group deposition and removal depend on metabolically derived substrates and cofactors (Etchegaray and Mostoslavsky, 2016). The availability of methyl donors is tightly controlled by one-carbon metabolic pathways, including the methionine cycle, folate cycle, and serine-glycine metabolism. These pathways converge on the generation of SAM, the universal methyl donor that supports DNA, histone, and RNA methylation (Ducker and Rabinowitz, 2017; Friso et al., 2017). Conversely, active demethylation is constrained by other metabolic variables: TET dioxygenases, Jumonji C-domain histone demethylases, FTO, and ALKBH5 depend on α-ketoglutarate, oxygen, and Fe^2+^, whereas LSD1/KDM1A uses FAD-dependent chemistry. Consequently, metabolic alterations can influence methylation landscapes by modulating both methylation writing and erasing reactions, although the strength of causal evidence varies across metabolic and methylation contexts.

Methylation remodeling can function as one mechanism through which metabolic conditions are translated into gene-regulatory and phenotypic outputs (Lu and Thompson, 2012). Across distinct regulatory layers, DNA, histone, and RNA methylation can influence chromatin accessibility, transcriptional programs, and RNA fate, thereby contributing to cell-state transitions in response to environmental stress (Artika et al., 2025). Evidence from perturbation studies in selected experimental systems, together with broader associative observations, indicates that metabolic rewiring can be accompanied by remodeling of DNA, histone, and RNA methylation in contexts linked to EMP, cancer stemness, immune escape, therapeutic resistance, and, in specific settings, tumor dormancy (Pavlova and Thompson, 2016; Davies et al., 2023; Perez-Gonzalez et al., 2023). These observations motivate a framework in which metabolically responsive methylation contributes, in a context-dependent manner, to the accessibility or stability of adaptive cancer cell states.

In this review, we propose that metabolic rewiring and methylation remodeling are functionally connected processes that can contribute to cancer cell plasticity. We refer to the resulting dynamic and context-dependent distribution of DNA, histone, and RNA methylation states across cancer cell populations as the “adaptive methylation landscape,” which can be remodeled in response to metabolic, microenvironmental, and therapeutic pressures and is associated with changes in the spectrum of phenotypic states accessible to cancer cells. This concept extends beyond static methylation heterogeneity by emphasizing perturbation-responsive, state-associated, and potentially reversible remodeling. We discuss how alterations in one-carbon metabolism reshape DNA, histone, and RNA methylation landscapes, how these methylation programs may initiate, permit, or stabilize cell-state transitions during tumor evolution, and how targeting metabolic-methylation circuits may provide new therapeutic opportunities for overcoming tumor adaptation and treatment resistance. We further examine natural compounds and natural-product derivatives as mechanistically heterogeneous modulators of the epigenetic–metabolic axis, alongside established and emerging methylation- and metabolism-directed therapies, while considering their translational limitations and potential unintended effects on cancer plasticity. By integrating metabolism, epigenetics, and cellular plasticity into a unified but testable framework, we aim to provide a comprehensive perspective on the mechanisms through which cancer cells continuously redefine their identity during disease progression.

This review was developed through a narrative, non-systematic assessment of literature. Relevant studies were identified primarily through PubMed and Web of Science using combinations of terms related to cancer plasticity, one-carbon metabolism, methionine metabolism, SAM, DNA methylation, histone methylation, m^6^A, methyltransferases, demethylases, epithelial–mesenchymal plasticity, cancer stemness, dormancy, lineage plasticity, therapy resistance, and natural compounds. Literature available through September 2026 was considered, without a prespecified start date. We prioritized mechanistic studies that directly linked metabolic perturbation to methylation changes and cancer-associated phenotypes, together with landmark studies establishing relevant methylation pathways and recent clinical or translational reports on therapeutic targeting. For the newly added discussion of natural compounds and natural-product derivatives, emphasis was placed on recent experimental studies providing direct evidence of methylation-related mechanisms rather than descriptive anticancer activity alone. Reference lists of key articles were additionally examined to identify relevant primary studies. Because this was not conducted as a systematic review or meta-analysis, study selection was designed to illustrate representative mechanistic relationships and emerging concepts rather than to provide exhaustive literature coverage or quantitative evidence synthesis.

## 2 Metabolic origins of cancer methylation landscapes

### 2.1 One-carbon metabolism as the methyl donor hub

The ability of cancer cells to dynamically remodel their methylation landscapes is fundamentally dependent on metabolic networks that generate and maintain intracellular methyl donor availability. Among these networks, one-carbon metabolism serves as the central biochemical hub linking nutrient availability to epigenetic regulation (Ducker and Rabinowitz, 2017; Friso et al., 2017). By integrating inputs from methionine, folate, serine, and glycine metabolism, one-carbon metabolism supplies the methyl groups required for DNA, histone, and RNA methylation, thereby establishing a direct connection between metabolic state and cell fate determination (Kaelin and McKnight, 2013). At the core of this network lies the methionine cycle, which functions as the primary source of the universal methyl donor, SAM. Methionine is converted to SAM by methionine adenosyltransferases (MATs), and SAM subsequently donates its methyl group to a wide range of methyltransferases, including DNA methyltransferases (DNMTs), histone methyltransferases, and RNA methyltransferases. Following methyl transfer, SAM is converted to S-adenosylhomocysteine (SAH), which is subsequently hydrolyzed to homocysteine. Homocysteine can then be remethylated to regenerate methionine, thereby completing the cycle and sustaining methyl donor availability (Ducker and Rabinowitz, 2017). The folate cycle provides the essential one-carbon units that support methionine regeneration and methyl donor homeostasis. Folate-derived intermediates participate in the transfer of one-carbon groups required for the conversion of homocysteine to methionine through methionine synthase-dependent reactions. Consequently, alterations in folate metabolism can profoundly influence intracellular SAM levels and methylation capacity (Lan et al., 2018). Notably, multiple enzymes involved in folate metabolism are aberrantly expressed across diverse cancer types, consistent with altered pathway capacity in malignant cells; however, expression changes alone do not establish altered metabolic flux (Nilsson et al., 2014; Dekhne et al., 2020).

An equally important contributor to one-carbon metabolism is the serine-glycine pathway. Serine serves as a major donor of one-carbon units through the action of serine hydroxymethyltransferases, generating glycine and one-carbon intermediates that feed into folate metabolism (Sun et al., 2023). Enhanced serine synthesis or uptake occurs in multiple tumor contexts and can support tumor growth and therapeutic adaptation. By fueling folate-mediated one-carbon transfer reactions, the serine-glycine pathway provides a continuous supply of one-carbon units that sustain folate metabolism, methionine regeneration, and ultimately SAM synthesis (Ducker and Rabinowitz, 2017).

Importantly, the biological significance of one-carbon metabolism extends beyond the generation of methyl donors (Dekhne et al., 2020). Emerging evidence suggests that cancer cells actively modulate one-carbon flux in response to nutrient availability, hypoxia, therapeutic stress, and microenvironmental cues. Such metabolic rewiring can alter intracellular SAM and SAH pools, thereby influencing the methylation potential of the cell (Mentch et al., 2015). Since methyltransferase activity depends on SAM availability, metabolic changes that alter the SAM/SAH balance can influence methylation reactions even in the absence of mutations in the methylation machinery. This close metabolic dependence makes methylation one of the epigenetic mechanisms most directly coupled to cellular metabolic state. Whereas chromatin regulators are often viewed as autonomous determinants of gene expression, their activity is intrinsically constrained by the availability of metabolic substrates. In this context, one-carbon metabolism functions not merely as a supplier of methyl groups but as one metabolic determinant of methylation capacity. By controlling the abundance and distribution of methyl donors, one-carbon metabolism contributes to setting the capacity of cancer cells to remodel DNA, histone, and RNA methylation programs in response to environmental challenges. Interpretation of nutrient-dependent metabolic effects *in vitro* also requires caution, because standard culture media often contain nutrient concentrations that differ from physiological conditions and can impose metabolic states that do not fully recapitulate the *in vivo* tumor environment. This limitation is particularly relevant when evaluating how methionine and folate availability influences one-carbon metabolism, methyl-donor availability, and downstream methylation responses (Vande Voorde et al., 2019).

### 2.2 Differential SAM sensitivity of methyltransferases

Changes in SAM availability are unlikely to affect all methylation reactions uniformly. The metabolic responsiveness of a methyltransferase depends in part on its biochemical relationship with SAM. Reported kinetic measurements indeed show substantial variation in SAM affinity among methyltransferases, although absolute values vary across experimental systems and should therefore be compared cautiously (Mentch and Locasale, 2016). However, SAM affinity alone does not determine the cellular response to methyl-donor limitation. The metabolic sensitivity of an individual methylation reaction can also depend on enzyme abundance and catalytic properties, substrate availability, subcellular context, and competition for the intracellular methyl-donor pool. In addition, S-adenosylhomocysteine (SAH), the product of SAM-dependent methylation reactions, can inhibit methyltransferase activity, making the SAM:SAH ratio an important determinant of cellular methylation potential rather than SAM concentration alone (Mentch et al., 2015; Mentch and Locasale, 2016). Thus, methyltransferases that use the same SAM pool can nevertheless exhibit distinct thresholds and kinetics in response to a common metabolic perturbation.

This differential metabolic sensitivity provides a potential mechanism through which one-carbon metabolism can selectively remodel methylation landscapes rather than simply causing global hyper- or hypomethylation. Experimental evidence is particularly strong for histone methylation. Methionine restriction alters intracellular SAM and SAH and produces rapid and reversible changes in histone methylation, with H3K4me3 showing pronounced metabolic responsiveness (Mentch et al., 2015). Genome-wide analyses further indicate that methionine restriction does not uniformly erase H3K4me3 across the genome; instead, the magnitude and genomic distribution of H3K4me3 changes are context dependent and can be associated with transcriptional programs linked to cell identity. Importantly, there is no basis to assume that an equivalent metabolic perturbation will produce comparable responses across DNA, histone, and RNA methylation, because each layer is governed by distinct enzymes, substrates, turnover rates, and opposing demethylation reactions. Thus, the consequences of altered methyl-donor availability depend not only on the abundance of SAM but also on the kinetic properties of the methylation machinery, genomic or molecular substrate context, and the regulatory state of the cell (Dai et al., 2018). Importantly, differential SAM sensitivity should not be interpreted as a fixed hierarchy in which DNA, histone, or RNA methylation as an entire system is uniformly more metabolically sensitive than another. Rather, metabolic responsiveness is enzyme-, mark-, and context-dependent.

Methyl-donor availability, however, represents only one side of the metabolic control of methylation. Net methylation states also depend on the opposing activity of demethylases, many of which are controlled by metabolic inputs distinct from SAM. TET DNA dioxygenases and Jumonji C-domain histone lysine demethylases require α-ketoglutarate (α-KG), molecular oxygen, and Fe^2+^ for catalytic activity, whereas the RNA m^6^A demethylases FTO and ALKBH5 belong to the same Fe^2+^/α-KG-dependent dioxygenase superfamily (Jia et al., 2011; Zheng et al., 2013). In addition, LSD1/KDM1A uses FAD as a redox cofactor, illustrating that even within the eraser arm, metabolic requirements are not uniform. Consequently, the methylation state of a cell reflects the balance between metabolically constrained writing and erasing reactions rather than methyltransferase activity alone (Figure 1; Table 1). This distinction is important for the adaptive methylation landscape framework because it predicts selective redistribution of methylation states under metabolic stress rather than uniform loss of methylation across the epigenome and epitranscriptome.

**FIGURE 1:**
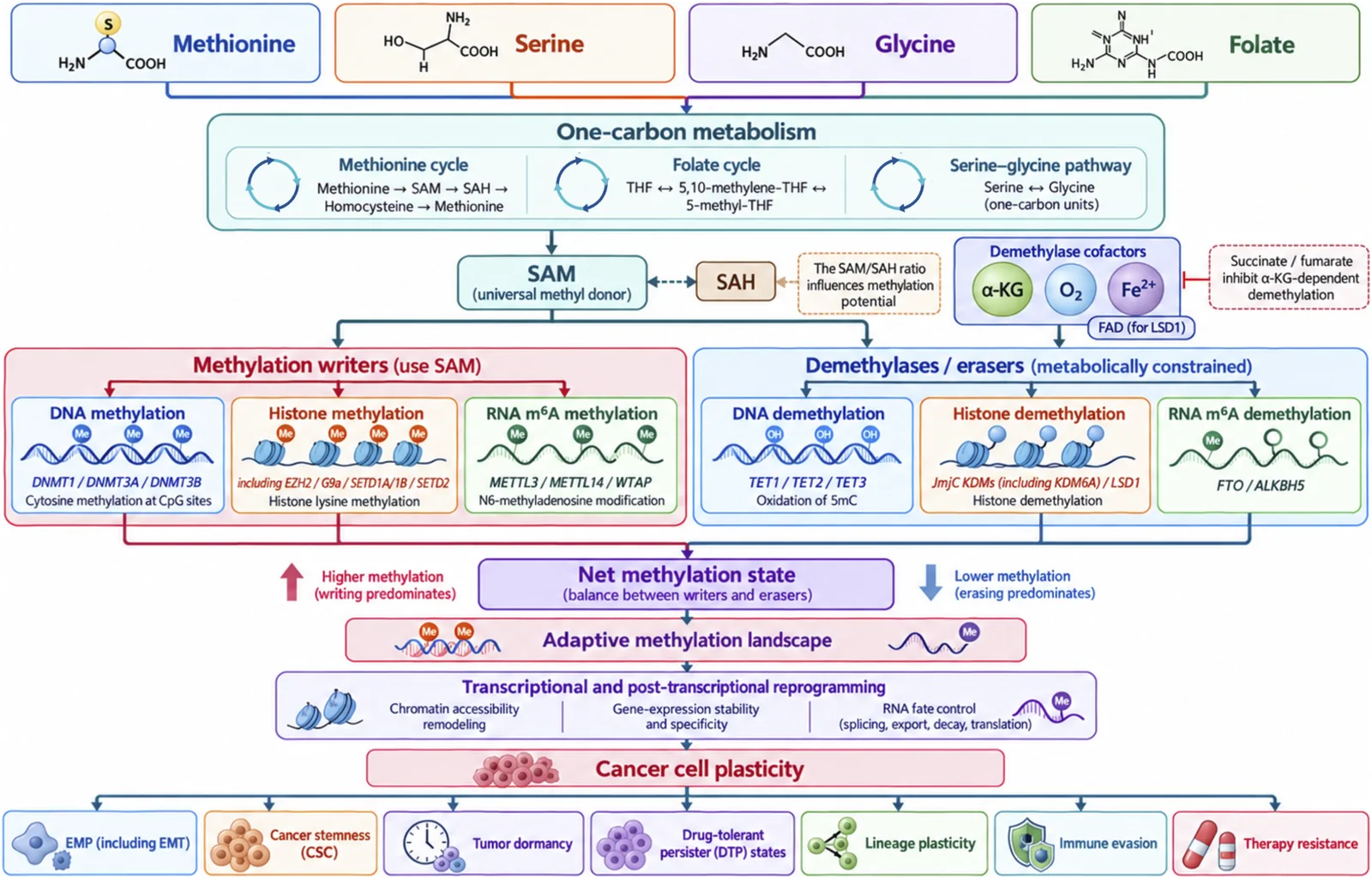
Metabolic control of methylation writer–eraser balance and cancer cell plasticity. Methionine, serine, glycine, and folate support one-carbon metabolism and regulate intracellular S-adenosylmethionine/S-adenosylhomocysteine (SAM/SAH) balance, thereby constraining SAM-dependent methylation writers, including DNA methyltransferases, histone methyltransferases, and the METTL3/METTL14/WTAP m6A writer complex. In parallel, DNA, histone, and m6A demethylation are regulated by metabolically sensitive erasers. TET enzymes, Jumonji C-domain histone demethylases, FTO, and ALKBH5 require α-ketoglutarate (α-KG), O_2_, and Fe^2+^, whereas LSD1/KDM1A uses flavin adenine dinucleotide (FAD)-dependent oxidative chemistry; succinate and fumarate can inhibit α-KG-dependent demethylation. The balance between methylation writing and erasing shapes context-dependent net methylation states that contribute to the adaptive methylation landscape. These states influence transcriptional and post-transcriptional reprogramming and thereby contribute to epithelial–mesenchymal plasticity (EMP), including epithelial–mesenchymal transition (EMT), cancer stemness, tumor dormancy, drug-tolerant persister (DTP) states, lineage plasticity, immune evasion, and therapy resistance.

**TABLE 1:** Representative methylation writers and erasers and their metabolic dependencies.

Methylation system	Representative enzyme(s)	Class	Principal substrate/mark	Metabolic substrate, cosubstrate, or cofactor dependence	SAM sensitivity/metabolic responsiveness	Key references (PMID)
DNA methylation	DNMT1	Writer	Maintenance of CpG 5mC, preferentially on hemimethylated DNA	SAM is the methyl donor and is converted to SAH	Directly SAM dependent; cellular methylation potential is influenced by SAM availability and the SAM/SAH ratio, but a defined physiological SAM-response threshold for DNMT1 has not been established	26647078; 27876555
DNA methylation	DNMT3A, DNMT3B	Writer	*De novo* CpG 5mC deposition	SAM → SAH	Directly SAM dependent; enzyme- and context-specific responses to physiological SAM fluctuations remain incompletely defined	26647078; 27876555
DNA methylation	TET1, TET2, TET3	Eraser pathway	Sequential oxidation of 5mC to 5hmC, 5fC, and 5caC, facilitating DNA demethylation	Require O_2_, α-KG, and Fe^2+^	Not directly SAM dependent; activity is sensitive to O_2_, α-KG, Fe^2+^, and metabolic/redox conditions. Hypoxia reduces TET activity, while succinate and fumarate can inhibit α-KG-dependent demethylation	24439369; 27533040; 22677546
Histone methylation	SETD1A/SETD1B; KMT2-family enzymes	Writer	H3K4 methylation, including H3K4me3	SAM → SAH	H3K4 methylation is particularly responsive to methionine/SAM availability in experimental systems; methionine restriction can selectively remodel H3K4me3 rather than uniformly depleting histone methylation	26411344; 29769529
Histone methylation	EZH2 (PRC2)	Writer	H3K27me1/2/3, particularly H3K27me3	SAM → SAH	SAM dependent; histone methyltransferases differ in SAM kinetics, supporting non-uniform responses of individual methylation reactions to altered methyl-donor availability	21564555; 26647078
Histone methylation	EHMT2/G9a	Writer	Predominantly H3K9me1/2	SAM → SAH	SAM dependent; biochemical measurements support enzyme-specific SAM kinetics rather than uniform sensitivity across histone methyltransferases	21564555; 26647078
Histone methylation	SETD2	Writer	H3K36me3	SAM → SAH	Directly SAM dependent; quantitative sensitivity of SETD2/H3K36me3 to physiological SAM fluctuations is less well established than that of H3K4 methylation	16118227; 26647078
Histone methylation	JmjC-domain KDMs, including KDM6A	Eraser	Site-selective lysine demethylation; KDM6A demethylates H3K27me2/3	Require O_2_, α-KG, and Fe^2+^	Not directly SAM dependent; activity responds to oxygen and α-KG availability. KDM6A directly senses oxygen, and succinate/fumarate can inhibit α-KG-dependent histone demethylases	30872525; 22677546
Histone methylation	KDM1A/LSD1	Eraser	Primarily H3K4me1/2; context-dependent H3K9me1/2 demethylation	Requires FAD as a redox cofactor	Not directly SAM dependent; provides a distinct FAD-dependent route linking histone demethylation to cellular redox metabolism	15620353
RNA m^6^A methylation	METTL3–METTL14–WTAP complex	Writer	Predominantly internal mRNA m^6^A	METTL3 catalyzes SAM-dependent methyl transfer; METTL14 primarily supports substrate recognition and complex function	Directly SAM dependent. Structural studies establish SAM binding at the METTL3 catalytic site; the extent to which physiological SAM fluctuations determine cellular m^6^A levels remains incompletely defined	27373337; 27281194; 33902106
RNA m^6^A methylation	FTO	Eraser	m^6^A and related methylated adenosine substrates, depending on RNA context	Requires O_2_, α-KG, and Fe^2+^	Not directly SAM dependent; catalytic activity is governed by the metabolic/cofactor environment required for Fe^2+^/α-KG-dependent oxidative demethylation	22002720; 24662220
RNA m^6^A methylation	ALKBH5	Eraser	m^6^A in RNA	Requires O_2_, α-KG, and Fe^2+^	Not directly SAM dependent; catalytic activity depends on dioxygenase cofactors, while hypoxia can additionally increase ALKBH5 expression through HIF-dependent transcriptional regulation in selected cancer contexts	23177736; 27001847

5mC, 5-methylcytosine; 5hmC, 5-hydroxymethylcytosine; 5 fC, 5-formylcytosine; 5caC, 5-carboxylcytosine; α-KG, α-ketoglutarate; FAD, flavin adenine dinucleotide; m^6^A, N^6^-methyladenosine; SAH, S-adenosylhomocysteine; SAM, S-adenosylmethionine.

### 2.3 Metabolic regulation of DNA methylation

DNA methylation represents one of the most extensively studied epigenetic mechanisms in cancer and plays a central role in regulating gene expression, chromatin organization, genomic stability, and cellular identity (Greenberg and Bourc’his, 2019). Although aberrant DNA methylation has traditionally been viewed primarily through alterations in DNMT expression or activity, metabolic state can also influence DNA methylation by altering methyl-donor availability and the activity of metabolically dependent demethylation pathways (Izzo et al., 2021). By controlling methyl donor availability and methylation potential, metabolic pathways can influence the establishment and maintenance of cancer-specific DNA methylation landscapes (Pan et al., 2021).

DNA methylation is catalyzed by a family of DNMTs, including DNMT1, DNMT3A, and DNMT3B. DNMT1 primarily functions as a maintenance methyltransferase, preserving methylation patterns during DNA replication, whereas DNMT3A and DNMT3B are responsible for *de novo* methylation. Despite their distinct biological functions, all DNMTs rely on SAM as the methyl donor for transferring methyl groups to cytosine residues within CpG dinucleotides (Lyko, 2018; Greenberg and Bourc’his, 2019). Consequently, fluctuations in intracellular SAM availability can directly influence DNA methylation capacity. Multiple cancers exhibit increased expression or activity of pathways involved in methionine metabolism and folate-mediated one-carbon metabolism, changes that are consistent with enhanced capacity to maintain SAM and methyl donor availability. However, such pathway-level alterations do not necessarily demonstrate increased metabolic flux unless supported by direct isotope-tracing or related flux measurements. These metabolic adaptations may therefore contribute to DNA methylation remodeling during tumor progression and environmental adaptation (Dai et al., 2020; Izzo et al., 2021). In this context, metabolic networks function as upstream regulators that determine the permissiveness of DNA methylation programs. In contrast to SAM, SAH is a potent endogenous inhibitor of SAM-dependent methyltransferases, including DNMTs. Accumulation of SAH can suppress methyl transfer reactions by competitively inhibiting SAM-dependent methyltransferases. Consequently, the cellular methylation potential is determined not only by SAM abundance but also by the balance between SAM and SAH. Alterations in the SAM/SAH ratio have been associated with widespread changes in DNA methylation patterns, highlighting the importance of metabolic homeostasis in epigenetic regulation (Huo et al., 2021; Nishiyama and Nakanishi, 2021). These observations suggest that cancer-associated DNA methylation abnormalities may arise not only from genetic alterations affecting DNMTs but also from metabolic perturbations that alter methyl donor utilization. Net DNA methylation, however, is not determined by DNMT activity alone. Active DNA demethylation is initiated by TET dioxygenases, which oxidize 5-methylcytosine and require α-ketoglutarate, molecular oxygen, and Fe^2+^ (Lio et al., 2020). Consequently, DNA methylation can also shift when the metabolic environment alters TET activity, even without a primary change in SAM availability. This writer–eraser balance is particularly relevant in hypoxic tumors and in cancers with altered tricarboxylic-acid-cycle metabolism (Thienpont et al., 2016).

Metabolic regulation of DNA methylation extends beyond global methylation changes and contributes to the selective remodeling of genomic regions associated with cancer cell identity. Altered methyl donor availability can influence promoter and enhancer methylation, thereby modulating chromatin accessibility and transcriptional programs governing cell-state transitions (Bell et al., 2016; Pistore et al., 2017). Such remodeling has been functionally implicated in EMT, stemness, and therapy resistance (Pistore et al., 2017; Galle et al., 2020), while enhancer methylation dynamics have been associated with broader cancer plasticity and metastatic progression (Bell et al., 2016).

### 2.4 Metabolic regulation of histone methylation

Compared with DNA methylation, histone methylation generally enables more rapid and context-dependent remodeling of chromatin architecture and transcriptional activity. By modifying specific lysine and arginine residues on histone tails, histone methylation establishes permissive or repressive chromatin states that influence gene accessibility and cellular identity. Increasing evidence indicates that histone methylation is sensitive to metabolic inputs, providing one route through which nutrient availability can influence chromatin state and cancer-cell phenotype (Dai et al., 2020; Sun et al., 2022). Histone methylation is dynamically determined by opposing methyltransferase and demethylase activities. Histone methyltransferases use SAM as the methyl donor, making methyl-group deposition sensitive to one-carbon metabolism and the SAM/SAH ratio (Mentch and Locasale, 2016). In contrast, most Jumonji C-domain histone demethylases require α-ketoglutarate, oxygen, and Fe^2+^, linking methyl-group removal to mitochondrial metabolism, oxygen availability, and iron homeostasis. Methionine-dependent changes in SAM/SAH availability can remodel selected histone methylation marks, including H3K4me3, at a genome-wide scale (Mentch et al., 2015; Dai et al., 2018). Accordingly, metabolic perturbation can alter histone methylation by changing either writing or the erasing arm, and the resulting direction of change is mark- and context-specific.

Among histone methyltransferases, enhancer of zeste homolog 2 (EZH2) is one of the most extensively studied regulators of cancer progression. As the catalytic component of the Polycomb Repressive Complex 2 (PRC2), EZH2 mediates trimethylation of histone H3 lysine 27 (H3K27me3), a repressive chromatin mark associated with transcriptional silencing. Elevated EZH2 activity is frequently observed in aggressive tumors and has been linked to stemness maintenance, lineage plasticity, metastatic progression, and therapy resistance (Gan et al., 2018; Sun et al., 2022).

Another key regulator is G9a, which primarily catalyzes mono- and dimethylation of histone H3 lysine 9. These modifications contribute to facultative heterochromatin formation and transcriptional repression (Padeken et al., 2022). Emerging evidence suggests that G9a plays important roles in hypoxic adaptation, EMT, and cancer stem cells (CSCs) maintenance. Through modulation of H3K9 methylation, G9a enables cancer cells to silence differentiation-associated genes while preserving plastic cellular states (Dong et al., 2012; Nachiyappan et al., 2022). As with EZH2, the activity of G9a is closely linked to intracellular methyl donor availability, highlighting the metabolic sensitivity of chromatin remodeling mechanisms (Poulard et al., 2021).

In contrast to the predominantly repressive functions of EZH2 and G9a, several SET-domain histone methyltransferases deposit activating methylation marks. SETD1A and SETD1B are COMPASS-family H3K4 methyltransferases that contribute to H3K4 methylation associated with transcriptionally active chromatin, whereas SETD2 is functionally distinct and serves as the principal H3K36 trimethyltransferase, contributing to transcriptional elongation, genome stability, and RNA processing. Dysregulation of these methyltransferases has been implicated in tumor evolution and adaptive transcriptional reprogramming. Through deposition of distinct activating histone marks, they can influence gene networks associated with proliferation, survival, and phenotypic flexibility (Zhao et al., 2021; Weng et al., 2024).

Histone demethylases provide a complementary metabolic control point. Jumonji C-domain lysine demethylases are α-KG-dependent dioxygenases whose catalytic activity requires molecular oxygen and Fe^2+^. KDM6A, which removes H3K27me2/3, is particularly sensitive to oxygen availability and can function as an oxygen-responsive chromatin regulator. Moreover, succinate and fumarate can competitively inhibit α-KG-dependent histone demethylases, thereby favoring retention of selected methylated histone states (Xiao et al., 2012; Chakraborty et al., 2019). Not all histone demethylases share this metabolic requirement: LSD1/KDM1A uses FAD-dependent oxidative chemistry and was originally identified as an H3K4me1/2 demethylase (Shi et al., 2004); in specific transcriptional contexts, it can also demethylate H3K9me1/2 (Metzger et al., 2005). These mechanistic differences further illustrate why changes in cellular metabolism need not produce uniform remodeling across the histone methylome.

Metabolic perturbations that alter the SAM/SAH balance or demethylase cofactor availability can reshape selected histone methylation states. These changes may in turn influence cell-state programs in a mark- and context-dependent manner. These observations support the concept that histone methylation serves as a metabolically responsive layer of chromatin regulation linking nutrient status to cellular identity.

### 2.5 m^6^A methylation and post-transcriptional plasticity

RNA methylation represents one of the most dynamic and rapidly reversible layers of epigenetic regulation. Among RNA methylation modifications, N6-methyladenosine (m^6^A) is the most extensively characterized in cancer and therefore represents the primary focus of this review. M^6^A provides a dynamic post-transcriptional regulatory layer that influences RNA stability, splicing, translation, and degradation. Increasing evidence suggests that m^6^A methylation serves as a highly responsive interface through which metabolic signals are translated into rapid changes in gene expression programs (Yang et al., 2020; Jiang et al., 2021). Consequently, m^6^A is increasingly recognized as a key determinant of cancer cell plasticity.

Like DNA and histone methylation, m^6^A deposition depends on SAM as the methyl donor. This metabolic dependency renders m^6^A deposition sensitive to one-carbon metabolism and cellular methyl donor availability. Because m^6^A deposition is SAM dependent, metabolic pathways that alter SAM production can influence m^6^A methylation. For example, mechanistic target of rapamycin complex 1 (mTORC1)-dependent stimulation of SAM synthesis has been shown to increase m^6^A-dependent mRNA regulation (Villa et al., 2021). The installation of m^6^A marks is primarily mediated by a core methyltransferase complex composed of METTL3, METTL14, and WTAP. METTL3 functions as the catalytic core responsible for methyl transfer, whereas METTL14 contributes to RNA substrate recognition and complex stability (Huang et al., 2022; Boulias and Greer, 2023). WTAP serves as a regulatory scaffold that facilitates localization and assembly of the methyltransferase machinery. Dysregulation of this writer complex has been implicated in multiple cancer types and is frequently associated with tumor progression, stemness maintenance, metastatic dissemination, and therapeutic resistance. Importantly, because m^6^A deposition relies on SAM-dependent methyl transfer reactions, metabolic changes that alter SAM availability can influence m^6^A deposition and the extent of RNA methylation (Deng et al., 2023; Liu J. et al., 2023). Conversely, m^6^A removal is mediated principally by the demethylases FTO and ALKBH5, both Fe^2+^/α-KG-dependent dioxygenases. Their distinct metabolic requirements provide an additional route through which the cellular metabolic environment can regulate net m^6^A abundance independently of changes in SAM supply (Jia et al., 2011; Zheng et al., 2013). In cancer cells, hypoxia can additionally increase ALKBH5 expression through hypoxia-inducible factor (HIF)-dependent signaling, demonstrating that the biological output of the eraser arm reflects both catalytic cofactor availability and regulation of enzyme abundance (Zhang C. et al., 2016). A notable feature of RNA methylation is its capacity to regulate cellular phenotypes on a timescale that is often more rapid than that of DNA methylation and many chromatin-based regulatory mechanisms. Whereas DNA methylation and histone methylation frequently establish long-term transcriptional states, m^6^A modifications can rapidly adjust transcript fate without requiring extensive chromatin remodeling (An and Duan, 2022). This property makes m^6^A particularly well suited for regulating adaptive cell-state transitions under fluctuating environmental conditions.

Other SAM-dependent RNA modifications, including 5-methylcytosine (m^5^C), N1-methyladenosine (m^1^A), N7-methylguanosine (m^7^G), and 2-O-methylation, are also increasingly implicated in cancer biology. However, their direct links to one-carbon metabolism and reversible cancer cell plasticity remain less systematically characterized than those of m^6^A. Accordingly, the present review focuses primarily on m^6^A while recognizing that broader RNA methylation may represent an additional layer of metabolic–epigenetic regulation.

The principal metabolic dependencies of representative DNA, histone, and RNA methylation writers and erasers are summarized in Table 1, highlighting that methylation states reflect not only shared dependence of writer enzymes on SAM but also enzyme-specific SAM sensitivity and distinct metabolic requirements of demethylases. Collectively, accumulating evidence supports a conceptual framework in which one-carbon metabolism provides a shared metabolic constraint on multiple methylation processes. Methionine-derived SAM serves as a common methyl donor for DNA, histone, and RNA methyltransferases, allowing changes in one-carbon metabolism to influence methylation across genomic, chromatin, and transcriptomic layers. However, shared dependence on SAM does not necessarily imply that these methylation systems constitute a uniformly coupled regulatory network. Rather, their responses to metabolic perturbation may differ according to methyltransferase kinetics, SAM and SAH sensitivity, substrate accessibility, and cellular context. Moreover, active demethylation is constrained by a distinct set of metabolic inputs. TET enzymes, JmjC-domain histone demethylases, FTO, and ALKBH5 depend on α-KG, oxygen, and Fe^2+^, whereas LSD1/KDM1A uses FAD-dependent oxidative chemistry. The accumulation of metabolites such as succinate and fumarate can further inhibit α-KG-dependent demethylases. Accordingly, the net methylation state of a cancer cell emerges from the balance between metabolically regulated writing and erasing reactions rather than from SAM availability alone. Direct molecular crosstalk has been established between selected epigenetic layers, particularly DNA and histone methylation, but comparable mechanistic coupling across DNA, histone, and RNA methylation has not yet been demonstrated as a general principle. We therefore view these processes as distinct but metabolically connected regulatory layers whose differential responses to a shared metabolic environment may collectively shape cancer cell identity and plasticity. These context-dependent net methylation states collectively constitute the biochemical basis of the adaptive methylation landscape proposed here, linking metabolic conditions to transcriptional and post-transcriptional reprogramming and, ultimately, to the repertoire of cancer cell states accessible under environmental and therapeutic pressure (Figure 1) (Box 1).

BOX 1 **Operational definition of the adaptive methylation landscape.**

Adaptive methylation landscape: A dynamic and context-dependent distribution of DNA, histone, and RNA methylation states across cancer cell populations that is coupled to defined metabolic, microenvironmental, or therapeutic pressures and is associated with reversible transitions among phenotypic states. Methylation heterogeneity alone is therefore insufficient to establish an adaptive methylation landscape. Operationally, an adaptive methylation landscape should be characterized by four features: (i) measurable heterogeneity in methylation states among cancer cell populations or states; (ii) reproducible redistribution of methylation-associated states after a defined metabolic, microenvironmental, or therapeutic perturbation; (iii) reversibility or further remodeling when the perturbation is removed or altered; and (iv) a corresponding change in phenotypic-state accessibility or stability. Evidence that perturbing the relevant methylation machinery modifies the associated cell-state transition would provide additional support for a causal role of methylation rather than simple correlation.

## 3 Methylation landscapes govern cancer cell state transitions

The causal relationship between methylation remodeling and cancer cell plasticity is context dependent rather than strictly unidirectional. In some settings, DNA methylation remodeling is required for the establishment or maintenance of plastic states, as demonstrated by DNMT perturbation studies in EMT models; in others, environmental or transcriptional signals initiate cell-state transitions and are accompanied by subsequent, reversible methylation remodeling (Cardenas et al., 2014; Pistore et al., 2017; Galle et al., 2020). Thus, methylation may function as an initiator, permissive mechanism, or stabilizer of plastic cell states, a distinction that is important when considering methylation-directed therapeutic intervention.

### 3.1 Epithelial-mesenchymal plasticity and metastasis

EMP encompasses reversible transitions along the epithelial–mesenchymal spectrum, including EMT and intermediate or hybrid epithelial–mesenchymal states, and contributes to metastasis, therapeutic resistance, and tumor evolution. This plasticity enables tumor cells to dynamically adapt to changing microenvironmental conditions during metastatic progression (Akhmetkaliyev et al., 2023; Haerinck et al., 2023).

DNA methylation contributes to EMT by stabilizing transcriptional programs. In nasopharyngeal carcinoma cells, DNMT1-dependent hypermethylation of the CDH1 promoter suppresses E-cadherin expression, providing a direct mechanism through which DNA methylation can reinforce loss of epithelial identity during EMT (Zhang R. L. et al., 2016). Methylation remodeling at regulatory regions associated with EMT transcriptional programs may facilitate mesenchymal gene expression (Pistore et al., 2017). At the chromatin level, histone methylation further reinforces these transitions. In gastric cancer, EZH2-mediated H3K27me3 promotes EMT and pluripotent-like phenotypes through repression of PTEN. In parallel, m^6^A RNA methylation provides a rapid and reversible layer of regulation by controlling the stability and translation of EMT-associated transcripts, allowing cancer cells to rapidly adjust gene expression in response to environmental stress (Garbo et al., 2021).

DNA, histone, and m^6^A methylation contribute complementary regulatory layers to the stability and flexibility of EMT programs. DNA methylation can stabilize longer-term transcriptional states, histone methylation modulates chromatin competence, and m^6^A methylation dynamically fine-tunes transcript fate (Haerinck et al., 2023). Through these complementary mechanisms, methylation remodeling can either facilitate the establishment of EMT-associated transcriptional programs or stabilize, and fine-tune states initially induced by transcription factor networks and microenvironmental signals. Such context-dependent regulation may support the maintenance of hybrid epithelial–mesenchymal states and their associated metastatic and therapeutic adaptability.

### 3.2 Cancer stemness

Among the diverse manifestations of cancer cell plasticity, the acquisition of stem-like properties represents one of the most clinically relevant adaptive states. CSCs possess self-renewal and tumor-initiating capacities and are closely associated with metastasis, therapeutic resistance, and disease recurrence.

Stemness is not necessarily confined to a fixed cell population; differentiated cancer cells may regain stem-like properties under hypoxia, nutrient stress, stromal signaling, or treatment pressure through reversible transcriptional and epigenetic reprogramming (Lee et al., 2025). RNA methylation provides several direct links between environmental stress and stemness. In breast cancer, hypoxia induces HIF-1α- and HIF-2α-dependent expression of the m^6^A demethylase ALKBH5, which removes m^6^A from NANOG mRNA, increases its stability, and promotes breast cancer stem-cell enrichment and tumor initiation (Zhang C. et al., 2016). ALKBH5 also supports leukemia stem-cell self-renewal in acute myeloid leukemia, although through different downstream targets. It maintains oncogenic transcripts such as TACC3, while the KDM4C-ALKBH5-AXL axis links histone demethylation, chromatin accessibility, and RNA methylation within the same stem-cell program (Shen et al., 2020; Wang et al., 2020). The m^6^A writer complex sustains malignant stemness through a distinct mechanism. METTL14 promotes AML initiation and leukemia stem-cell maintenance by regulating MYB and MYC transcripts, whereas METTL3 supports an undifferentiated leukemic state through enhanced expression of MYC, BCL2, PTEN, and other leukemia-associated proteins. Depletion of either writer promotes differentiation and impairs leukemia maintenance (Vu et al., 2017; Weng et al., 2018). m^6^A readers further determine the fate of modified transcripts. YTHDF2 promotes the decay of selected m^6^A-containing RNAs required for leukemia stem-cell fitness; its depletion activates pro-apoptotic programs and suppresses leukemia initiation while largely preserving normal hematopoietic stem cells (Paris et al., 2019). Histone methylation provides a more stable layer of regulation. In colorectal cancer, G9a-dependent H3K9 methylation supports pluripotency-associated transcriptional programs, organoid renewal, and tumor initiation, whereas genetic or pharmacological inhibition of G9a reduces these stem-like properties (Bergin et al., 2021). These methylation circuits remain metabolically constrained because DNA, histone, and RNA methyltransferases require SAM. Changes in methionine uptake, one-carbon flux, or SAM synthesis may therefore influence CSC maintenance, although methionine restriction also affects redox balance, nucleotide synthesis, and translation. Its effects should not be attributed exclusively to methylation without direct analysis of SAM/SAH and the relevant methylation marks. Cancer stemness is therefore maintained through target-specific and context-dependent methylation programs rather than a uniform increase or decrease in global methylation. RNA methylation rapidly adjusts the stability and translation of stemness-related transcripts, whereas DNA and histone methylation help preserve chromatin states compatible with self-renewal and restricted differentiation.

### 3.3 Tumor dormancy

Tumor dormancy is a reversible state in which disseminated or residual cancer cells remain viable but non-proliferative, sometimes for years, before re-entering the cell cycle and causing metastatic relapse (Zhra et al., 2025; Kim, 2026). Dormant cells retain the capacity to resume growth, suggesting that their phenotype is maintained by reversible transcriptional and epigenetic programs (Turna and Demokan, 2026). NR2F1 is one of the best-characterized regulators of this state (Sosa et al., 2015). In disseminated tumor cells, NR2F1 activates SOX9-and retinoic acid receptor-associated programs, promotes quiescence, and contributes to chromatin repression compatible with long-term survival. Pharmacological activation of NR2F1 similarly induces growth arrest and suppresses metastatic outgrowth in head and neck cancer models, supporting a functional role for this pathway in dormancy maintenance (Khalil et al., 2022). DNA methylation can also influence whether dormant programs remain accessible. Combined treatment with the DNA-demethylating agent 5-azacytidine and retinoic acid restores TGF-β-SMAD4 signaling, induces an NR2F1-positive non-proliferative state, and limits metastatic development in head and neck and breast cancer models. This result suggests that selective DNA demethylation can re-establish dormancy-associated signaling rather than simply producing global transcriptional activation (Singh et al., 2023). Although *bona fide* tumor dormancy and therapy-induced drug-tolerant persister (DTP) states both involve reversible growth restraint, they are biologically distinct and should not be conflated. Direct evidence linking one-carbon metabolism or SAM availability to the establishment and maintenance of *bona fide* tumor dormancy remains limited; metabolic control of dormancy-associated methylation should therefore be regarded as an emerging Frontier rather than an established mechanism. DTP states, discussed separately in Section 4.4, provide a related setting in which reversible epigenetic remodeling and metabolic adaptation are better documented.

### 3.4 Lineage plasticity

Lineage plasticity represents one of the most profound manifestations of cancer cell plasticity, allowing tumor cells to abandon their original lineage identity and adopt alternative differentiation states under therapeutic pressure. Lineage plasticity involves large-scale reprogramming of cellular identity and has emerged as a major mechanism of acquired therapeutic resistance and tumor evolution (Davies et al., 2023; Pitarresi and Stanger, 2024). This phenomenon is particularly evident in lung and prostate cancers (Blee and Huang, 2019; Banerjee et al., 2025). In EGFR-mutant non-small cell lung cancer (NSCLC), prolonged tyrosine kinase inhibitor (TKI) treatment can induce histological transformation into small cell lung cancer (SCLC), whereas androgen receptor (AR)-targeted therapy may promote the emergence of neuroendocrine prostate cancer (Meng et al., 2025; Huo et al., 2026). These lineage transitions arise through an interplay between genetic alterations and epigenetic reprogramming. Genetic events such as RB1 and TP53 loss can establish a permissive background for lineage plasticity, whereas dynamic chromatin and methylation remodeling may facilitate the acquisition and stabilization of alternative cell identities under therapeutic pressure (Niederst et al., 2015; Ku et al., 2017).

Lineage switching can involve extensive epigenetic and methylation remodeling that contributes to the suppression of lineage-defining transcriptional programs and the stabilization of alternative developmental identities (Davies et al., 2023). DNA methylation contributes to the stabilization of newly acquired lineage-specific transcriptional programs, whereas EZH2-mediated H3K27me3 reshapes chromatin to enhance developmental competence (Shi et al., 2023). Therapy-induced metabolic rewiring may further facilitate these processes by influencing one-carbon metabolism and cellular methylation capacity. Together, these observations position lineage plasticity as a context in which metabolic and methylation remodeling may expand the repertoire of cancer cell identities under therapeutic pressure.

Collectively, EMT, stemness, dormancy, and lineage plasticity illustrate how context-dependent remodeling of DNA, histone, and m^6^A methylation can contribute to the establishment or stabilization of plastic cancer cell states. These regulatory layers need not change in parallel; rather, their relative contribution depends on the cell state, metabolic context, and selective pressure. Such state-dependent methylation remodeling provides one mechanistic route through which metabolic adaptation may influence phenotypic flexibility during tumor evolution.

## 4 Methylation-mediated plasticity in tumor adaptation

### 4.1 Adaptation to hypoxia

Hypoxia is a pervasive feature of solid tumors and promotes invasive behavior, stemness, metastatic dissemination, and treatment resistance through coordinated metabolic and epigenetic adaptation (Lee et al., 2020). One direct mechanism involves oxygen-dependent DNA demethylation. TET enzymes require molecular oxygen and α-ketoglutarate to oxidize 5-methylcytosine, and reduced oxygen availability suppresses TET activity without necessarily changing TET expression. This leads to locus-specific DNA hypermethylation and transcriptional silencing of genes involved in cell adhesion and tumor suppression, providing a route through which transient hypoxia can produce more durable changes in cancer-cell behavior (Thienpont et al., 2016). Histone demethylases also respond directly to oxygen tension. KDM6A is particularly oxygen sensitive, and hypoxia limits its removal of H3K27me3, thereby maintaining repressive chromatin at selected differentiation-associated genes. Loss of KDM6A produces similar effects, indicating that oxygen availability can alter lineage and differentiation programs through direct control of histone demethylase activity (Chakraborty et al., 2019). Hypoxia also regulates RNA methylation through HIF-dependent changes in m^6^A enzymes. In breast cancer cells, HIF-1α and HIF-2α induce ALKBH5, which removes m^6^A from NANOG mRNA, stabilizes the transcript, and expands the breast cancer stem-cell population. This pathway connects oxygen sensing with rapid post-transcriptional regulation of a defined plasticity-associated gene (Zhang C. et al., 2016).

The effects of hypoxia on methylation are also shaped by metabolic cofactors. Changes in mitochondrial activity, α-ketoglutarate production, succinate and fumarate accumulation, and serine–glycine one-carbon flux can modify the balance between methylation and demethylation reactions. In particular, succinate and fumarate can competitively inhibit α-KG-dependent dioxygenases, providing a direct mechanism through which altered mitochondrial metabolism can favor retention of selected DNA and histone methylation marks (Xiao et al., 2012). The resulting response is therefore locus- and enzyme-specific, with DNA, histone, and RNA methylation changing in different directions according to tumor lineage, oxygen duration, and nutrient availability (Lee et al., 2020; Shi et al., 2021). Hypoxia-associated methylation remodeling may contribute to transcriptional programs linked to stemness, invasion, and stress tolerance (Zhang et al., 2022). Hypoxic adaptation should therefore be viewed as the combined result of direct oxygen sensing by demethylases, HIF-dependent regulation of methylation enzymes, and metabolic changes in the availability of methyl donors and cofactors.

### 4.2 Adaptation to nutrient stress

Nutrient limitation is a defining feature of the tumor microenvironment and a major driver of cancer cell adaptation. Fluctuating supplies of methionine, glucose, and other metabolic substrates impose strong selective pressure, requiring tumor cells to continuously reprogram their metabolism. Methionine availability is particularly important because it determines intracellular SAM production and cellular methylation capacity. Methionine restriction reduces methyl-donor availability and can influence multiple SAM-dependent methylation reactions, although the magnitude and direction of the response may differ among DNA, histone, and RNA methylation systems. Nutrient-responsive signaling can also regulate one-carbon metabolism; for example, mTORC1 promotes SAM synthesis and m^6^A-dependent protein synthesis through regulation of methionine metabolism (Villa et al., 2021). Dietary serine and glycine restriction likewise perturbs one-carbon metabolism and can modify tumor responses to therapy (Maddocks et al., 2017).

A related feature of tumor metabolism is the so-called Hoffman effect, which describes the dependence of many cancer cells on exogenous methionine despite their apparent capacity to synthesize methionine endogenously (Kaiser, 2020). This methionine dependence has been linked to increased demand for transmethylation reactions and altered methionine-cycle flux, making extracellular methionine availability particularly relevant to tumor growth and epigenetic regulation (Hoffman, 2019). In this context, methionine restriction may affect cancer cells not only by lowering SAM availability and methylation potential but also by exploiting a broader metabolic vulnerability associated with methionine dependence. However, the extent of this dependence varies across tumor types and experimental systems, and its therapeutic exploitation must be balanced against the requirement for methionine in normal tissues.

Nutrient stress can induce selective remodeling across DNA, histone, and m^6^A methylation, with each layer contributing differently to metabolic adaptation, stress tolerance, and reversible cell-state transitions (An and Duan, 2022). By maintaining methylation homeostasis despite fluctuating nutrient availability, cancer cells establish metabolically responsive epigenetic programs that support long-term survival and adaptation.

### 4.3 Immune evasion

Immune surveillance creates strong selective pressure on tumor cells, and methylation-dependent regulation can determine whether cancer cells remain visible to cytotoxic lymphocytes (Lv et al., 2023; Hua et al., 2024). PRC2-mediated H3K27 trimethylation provides a direct mechanism for suppressing antigen presentation. Genome-wide screening identified PRC2 as a repressor of the major histocompatibility complex class I (MHC-I) antigen-processing and presentation pathway, with EZH2-dependent chromatin silencing reducing the expression of genes involved in peptide processing, β2-microglobulin assembly, and MHC-I surface presentation. Genetic or pharmacological disruption of PRC2 restores antigen-presentation programs and increases tumor-cell recognition by cytotoxic T cells in responsive models (Burr et al., 2019; Dersh et al., 2021).

H3K9 methylation controls a separate layer of tumor immunogenicity. KDM5B recruits the H3K9 methyltransferase SETDB1 to endogenous retroelements, maintaining their transcriptional silencing and limiting the production of immunostimulatory RNA and retroelement-derived antigens. KDM5B or SETDB1 depletion activates innate and adaptive immune responses and improves sensitivity to immune-checkpoint blockade in preclinical models (Zhang et al., 2021). DNA methylation similarly suppresses endogenous retroviral sequences, cancer-testis antigens, and interferon-responsive genes. DNMT inhibition can reactivate these normally silent regions, generate double-stranded RNA, and trigger a viral-mimicry response that enhances tumor-intrinsic immune signaling (Huang W. et al., 2024). The functional outcome depends on intact nucleic-acid sensing, interferon signaling, and antigen-presentation machinery, which may explain the variable immune effects of hypomethylating agents among tumors.

Metabolic competition can reshape immune-cell methylation outside the cancer cell itself (Gao et al., 2024). Tumor-cell uptake of extracellular methionine reduces methionine and SAM availability in neighboring T cells, leading to loss of H3K79me2 and impaired expression of genes required for effector function. Cancer-cell SLC43A2 depletion or methionine restoration can recover T-cell methylation and antitumor activity, providing direct evidence that tumor metabolism can regulate immune escape through methyl-donor competition (Bian et al., 2020; 2026). A related study showed that methionine consumption by cancer cells reduces SAM and H3K79me2 in CD4^+^ T cells, suppresses AMPK expression, and increases PD-1-associated dysfunction; methionine or SAM supplementation partially reverses this phenotype (Pandit et al., 2023).

These findings place immune evasion within a multilayered methylation network that regulates antigen presentation, retroelement silencing, interferon signaling, and T-cell fitness. The relevant methylation event may occur in tumor cells or immune cells, making the metabolic–methylation axis a tissue-wide regulator of antitumor immunity.

### 4.4 Therapy-induced drug-tolerant persister states

Anticancer therapy can induce reversible DTP states that enable a small fraction of tumor cells to survive otherwise lethal treatment without initially acquiring stable genetic resistance. Unlike lineage switching, which involves the acquisition of an alternative cellular identity, DTP states are characterized by transient and reversible phenotypic adaptation and can provide a reservoir from which genetically or epigenetically stabilized resistance subsequently emerges (Sharma et al., 2010; Marine et al., 2020). Although DTP states share reversible growth restraint with tumor dormancy, they arise under therapeutic selection and are considered separately here because metabolic–epigenetic coupling is better documented in this context.

Reversible chromatin remodeling is a defining feature of DTP formation. In EGFR-mutant cancer cells, KDM5A-dependent histone demethylation contributes to a transient drug-tolerant chromatin state that can revert following drug withdrawal (Sharma et al., 2010). More broadly, therapeutic stress can alter mitochondrial activity, nutrient utilization, redox balance, and one-carbon metabolism, thereby altering metabolites that regulate methylation and demethylation. Such changes may facilitate transient survival states, although the direct contribution of methyl-donor availability to DTP establishment remains incompletely defined.

DTP states therefore illustrate how reversible epigenetic remodeling can support survival during therapeutic stress without requiring immediate fixation by new genetic alterations. Longitudinal profiling before treatment, during the persister state, and after drug withdrawal or resistance acquisition will be particularly important for determining which methylation changes are transient adaptive responses and which become stabilized during the evolution of durable resistance.

## 5 Therapeutic opportunities

Therapeutic intervention in the epigenetic–metabolic axis can occur at multiple levels, including direct targeting of DNA, histone, or RNA methylation machinery, modulation of one-carbon metabolism and methyl-donor availability, and the use of natural compounds or natural-product derivatives that affect methylation writers, erasers, or their metabolic regulation (Figure 2). These strategies differ substantially in mechanistic specificity, clinical maturity, and safety profile, and their therapeutic effects are likely to depend on tumor context, metabolic vulnerability, and the adaptive cell states present.

**FIGURE 2:**
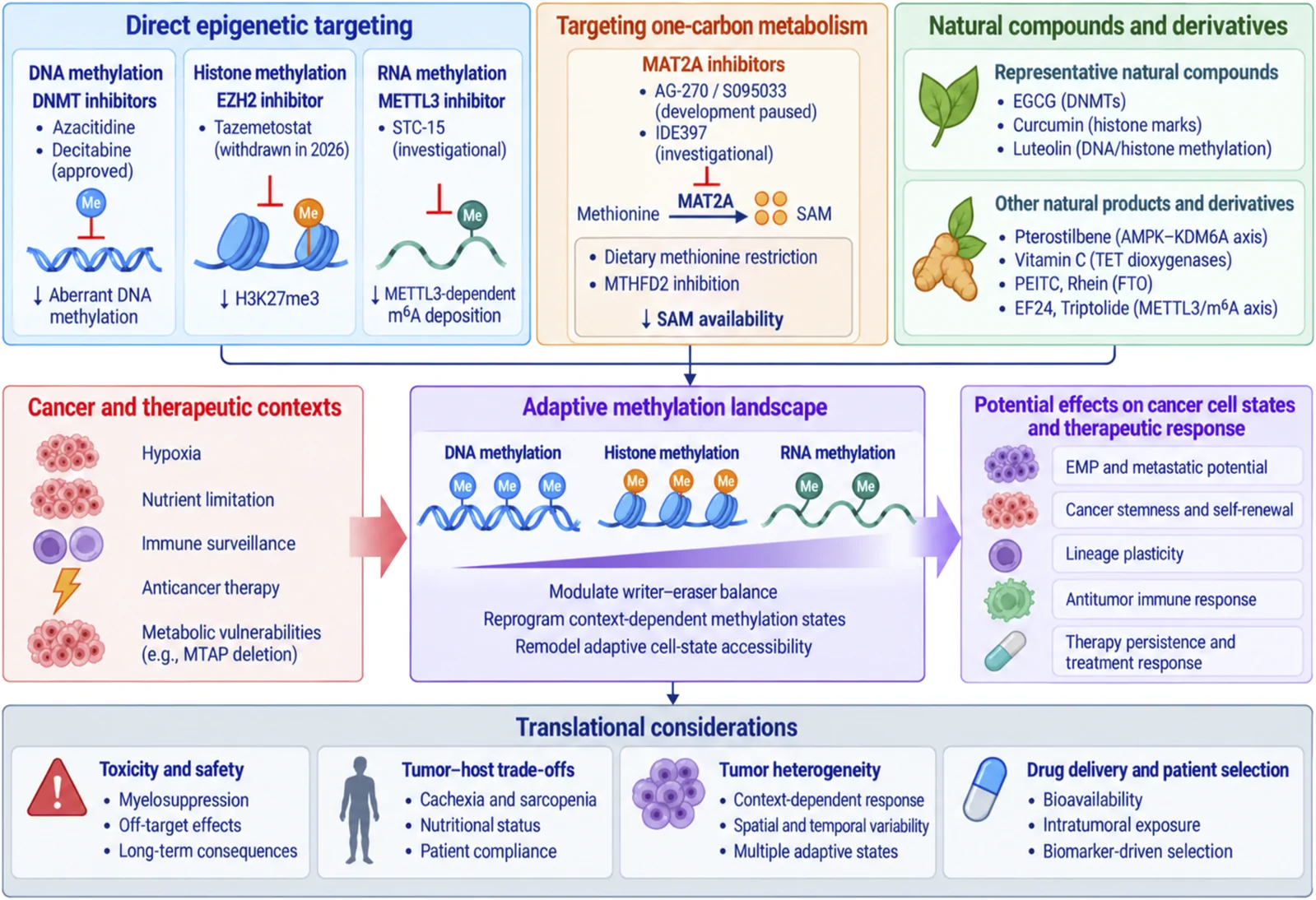
Therapeutic strategies targeting the cancer epigenetic–metabolic axis. Therapeutic intervention in the cancer epigenetic–metabolic axis can occur through several mechanistically distinct approaches. Direct epigenetic strategies include DNA methyltransferase (DNMT) inhibitors such as azacitidine and decitabine, inhibition of EZH2-mediated histone methylation, and emerging METTL3-directed approaches targeting RNA m6A methylation. Upstream metabolic strategies include inhibition of methionine adenosyltransferase 2A (MAT2A), dietary methionine restriction, and targeting of folate-dependent one-carbon metabolism, which can alter S-adenosylmethionine (SAM) availability and thereby influence SAM-dependent methylation reactions. Natural compounds and natural-product derivatives can modulate multiple components of this axis, including DNMTs, histone methyltransferases and demethylases, TET dioxygenases, and m6A writers or erasers. These interventions may reshape context-dependent methylation states and thereby influence cancer cell plasticity, including epithelial–mesenchymal plasticity, stemness, immune evasion, therapy persistence, and treatment response. Their clinical translation is constrained by differences in target specificity and developmental maturity, systemic toxicity, host–tumor metabolic trade-offs, tumor heterogeneity, pharmacokinetic limitations, and the potential for broad epigenetic perturbation to enable alternative adaptive cell states.

### 5.1 DNMT inhibitors

If DNA methylation stabilizes adaptive cancer cell states, disrupting DNA methylation offers an attractive strategy to limit cancer cell plasticity. Beyond reversing aberrant DNA methylation, DNMT inhibitors remodel methylation landscapes and destabilize epigenetic programs associated with stemness, EMP, immune evasion, and therapy resistance (Jones et al., 2019; Esteller et al., 2024). The nucleoside analogs azacitidine and decitabine are the first clinically approved DNMT inhibitors and remain the foundation of epigenetic therapy in hematologic malignancies. By trapping DNMT enzymes during DNA replication, these agents induce progressive passive DNA demethylation and restore the expression of silenced tumor suppressor, differentiation-associated, and immune regulatory genes. Recent studies suggest that the therapeutic effects of DNMT inhibitors extend beyond gene reactivation. Remodeling of DNA methylation landscapes can reduce stemness, impair EMP, and enhance antitumor immunity by restoring antigen presentation and interferon-responsive pathways. These findings provide a strong rationale for combining DNMT inhibitors with immune checkpoint blockade and other anticancer therapies (Jones et al., 2019; Yang J. et al., 2023).

Clinical experience also illustrates both the therapeutic value and limitations of this approach. In the randomized trial of higher-risk myelodysplastic syndromes (MDS), azacitidine prolonged median overall survival to 24.5 months compared with 15.0 months with conventional care regimens (hazard ratio, 0.58; 95% CI, 0.43–0.77), whereas a randomized phase III study of decitabine in MDS reported an overall response rate of 17% compared with 0% with supportive care. Thus, although DNMT-directed hypomethylating therapy can provide clinically meaningful benefit, responses are not universal and are often incomplete or non-durable (Fenaux et al., 2009). Additional efficacy and safety data are summarized in Table 2.

**TABLE 2:** Representative therapeutic strategies targeting the cancer epigenetic–metabolic axis.

Therapeutic strategy	Representative agent/intervention	Target and mechanism	Clinical status/biomarker context	Representative human efficacy/pharmacodynamics	Principal safety issue/limitation	Key reference(s)
DNA methylation targeting	Azacitidine (5-azacytidine)	Cytidine analogue that traps and depletes DNMTs after incorporation into nucleic acids, producing DNA hypomethylation at lower exposures.	FDA-approved hypomethylating agent; established therapy for myelodysplastic syndromes	Higher-risk MDS (AZA-001): median OS 24.5 vs 15.0 months with conventional care; HR 0.58.	Myelosuppression (neutropenia, thrombocytopenia, anemia) and gastrointestinal toxicity; responses are not universal or uniformly durable.	19230772
DNA methylation targeting	Decitabine (5-aza-2′-deoxycytidine)	Deoxycytidine analogue incorporated into DNA; covalently traps DNMTs, leading to DNMT depletion and passive DNA hypomethylation.	FDA-approved hypomethylating agent for MDS/CMML within the approved label	Randomized phase III MDS trial: ORR 17% vs 0% with supportive care; median response duration 10.3 months.	Myelosuppression and infection risk, including neutropenia, thrombocytopenia, anemia and febrile neutropenia.	16532500
Histone methylation targeting	Tazemetostat	Selective EZH2 inhibitor that suppresses PRC2-mediated H3K27 trimethylation.	Former U.S. accelerated approvals for epithelioid sarcoma and selected relapsed/refractory follicular lymphoma	Phase II follicular lymphoma: ORR 69% in EZH2-mutant and 35% in EZH2-wild-type cohorts. Epithelioid sarcoma: ORR 15%.	Risk of hematologic second primary malignancies prompted voluntary U.S. market withdrawal; historical efficacy must therefore be interpreted in the context of revised safety assessment.	33035457; 33035459
RNA m6A targeting	STC-15	Oral METTL3 inhibitor; decreases mRNA m6A and is associated with activation of dsRNA/innate immune signaling.	Investigational; first-in-human phase I study active, not recruiting. No approved indication.	ASCO 2024 interim data: 27 response-evaluable patients; ORR 11% (3 PRs), DCR 63%. Average 63% reduction in peripheral-blood mRNA m6A at 60 mg demonstrated target engagement.	Clinical dataset remains early and small; thrombocytopenia, rash and pruritus were among reported treatment-related events; long-term consequences of systemic METTL3 inhibition remain uncertain.	NCT05584111
One-carbon metabolism targeting	AG-270 / S095033	Oral MAT2A inhibitor that lowers SAM synthesis and exploits enhanced MAT2A dependence in MTAP-deficient tumors.	Investigational; first-in-human phase I trial completed in advanced tumors with CDKN2A/MTAP loss or MTAP-protein loss. Development of this compound was subsequently paused/discontinued.	n=40: maximal plasma SAM reductions 54–70%; paired tumor biopsies showed reduced SDMA. Two PRs; five additional patients had confirmed SD ≥16 weeks (16-week disease-control rate 17.5%).	Treatment-related fatigue, hyperbilirubinemia/bilirubin increase, anemia and thrombocytopenia; grade ≥3 treatment-related AEs occurred in 17.5%.	39762248
One-carbon metabolism targeting	IDE397	Selective oral MAT2A inhibitor intended to reduce SAM production in MTAP-deleted tumors.	Investigational; phase I study in MTAP-deleted solid tumors; active, not recruiting as of April 2026. No approved indication.	Preliminary monotherapy expansion data at the recommended dose have reported confirmed responses in MTAP-deleted NSCLC and urothelial cancer; estimates remain non-peer-reviewed and should be considered provisional.	Clinical efficacy and safety remain under evaluation; current response estimates derive from interim sponsor-reported datasets rather than a mature peer-reviewed efficacy publication.	NCT04794699
One-carbon metabolism targeting	Dietary methionine restriction	Reduces exogenous methionine availability and can lower systemic methionine/SAM supply, imposing a metabolic constraint on methionine-dependent tumors.	Experimental / early clinical evaluation only; no approved anticancer indication.	Small early clinical studies established feasibility and systemic methionine reduction, but no validated anticancer response magnitude or survival benefit has been established.	Adherence and nutritional adequacy are major limitations; prolonged restriction may aggravate weight loss, sarcopenia or cancer cachexia in susceptible patients.	12416254; 39729999

DCR, disease-control rate; DNMT, DNA methyltransferase; EZH2, enhancer of zeste homolog 2; MAT2A, methionine adenosyltransferase 2A; MDS, myelodysplastic syndrome; METTL3, methyltransferase-like 3; m6A, N6-methyladenosine; MTAP, methylthioadenosine phosphorylase; NSCLC, non-small-cell lung cancer; ORR, objective response rate; OS, overall survival; PR, partial response; PRC2, polycomb repressive complex 2; SAM, S-adenosylmethionine; SD, stable disease; SDMA, symmetric dimethylarginine.

DNMT inhibitors represent the first generation of therapies aimed at remodeling cancer methylation landscapes. Rather than functioning solely as DNA-demethylating agents, they should be viewed as modulators of adaptive methylation landscapes that can alter the accessibility and stability of cancer cell state transitions (Ghasemi, 2020).

### 5.2 Histone methyltransferase inhibitors

Histone methyltransferase inhibitors provide a promising strategy to disrupt the chromatin plasticity that supports adaptive cancer cell-state transitions. Among these agents, inhibitors targeting EZH2, the catalytic component of PRC2, are the most extensively investigated. By reducing H3K27me3 deposition, EZH2 inhibitors restore differentiation-associated gene expression and remodel chromatin states that sustain cancer cell plasticity (Liu R. et al., 2023). Accumulating evidence indicates that EZH2 inhibition suppresses stemness, EMP, lineage switching, and therapy resistance by destabilizing adaptive chromatin landscapes (Jin and Jeong, 2023; Yang X. et al., 2023). Tazemetostat, a selective EZH2 inhibitor, has provided clinical proof of concept for targeting histone methylation in both hematologic and solid malignancies. In a phase II study of relapsed or refractory follicular lymphoma, the objective response rate was 69% (95% CI, 53–82) in patients with EZH2-mutant tumors and 35% (95% CI, 23–49) in those with EZH2-wild-type tumors (Morschhauser et al., 2020). In advanced epithelioid sarcoma, tazemetostat achieved an objective response rate of 15% (95% CI, 7–26) in a phase II basket study (Gounder et al., 2020). These indications previously received accelerated approval in the United States; however, both approvals were voluntarily withdrawn in June 2026 following evidence of an increased risk of hematologic second primary malignancies. This regulatory development underscores the need to balance the therapeutic potential of epigenetic targeting with long-term safety considerations. Additional efficacy and safety information is summarized in Table 2. Beyond their effects on tumor cell identity, EZH2 inhibitors also enhance antitumor immunity by promoting antigen presentation and interferon-responsive gene expression, providing a strong rationale for combination with immune checkpoint blockade (Kang et al., 2020).

Despite these advances, targeting EZH2 alone is unlikely to suppress all forms of methylation-associated plasticity, because DNA, histone, and RNA methylation represent distinct regulatory layers that can respond differently to the same metabolic or therapeutic pressure (Xu et al., 2025). Consequently, the greatest therapeutic potential of EZH2 inhibitors may lie in combination strategies that target complementary mechanisms sustaining adaptive cell states.

### 5.3 RNA methylation-targeted therapies

Targeting RNA methylation has emerged as a promising strategy to disrupt the rapid and reversible adaptive responses that underlie cancer cell plasticity. Compared with DNA and histone methylation, m^6^A methylation provides a more rapid and reversible layer of post-transcriptional regulation, making the m^6^A machinery an attractive therapeutic target (Guan and Wong, 2024). Among current targets, METTL3 has received the greatest attention. Pharmacological inhibition of METTL3 reduces m^6^A deposition, alters RNA stability and translation, and suppresses transcriptional programs that support tumor growth, stemness, and therapeutic resistance (Moroz-Omori et al., 2021; Jiao et al., 2025). STC-15, an orally bioavailable small-molecule inhibitor of METTL3, has entered first-in-human clinical evaluation in patients with advanced malignancies (NCT05584111), indicating that clinical translation of METTL3 inhibition has begun. However, METTL3 inhibition remains investigational, no approved indication currently exists, and mature peer-reviewed data defining its antitumor efficacy, durability of response, and long-term safety remain limited. A unique advantage of RNA methylation-targeted therapies is their ability to interfere with rapid phenotypic adaptation. By limiting dynamic regulation of RNA fate, METTL3 perturbation has been shown to affect cancer stemness and treatment response in multiple preclinical models, although the direction and magnitude of these effects are highly context dependent and reflect the coordinated actions of m6A writers, erasers, and readers (Zeng et al., 2020). Moreover, because m^6^A methylation represents only one of several regulatory layers contributing to cancer cell plasticity, targeting METTL3 alone may not be sufficient to suppress all adaptive cell-state transitions. DNA and histone methylation, as well as other transcriptional and post-transcriptional mechanisms, may provide parallel routes through which cancer cells maintain phenotypic adaptability. Future therapeutic strategies will therefore likely rely on combination approaches that simultaneously disrupt multiple layers of the adaptive methylation landscape.

### 5.4 Targeting one-carbon metabolism

Targeting one-carbon metabolism offers an upstream strategy to simultaneously influence DNA, histone, and RNA methylation by limiting intracellular SAM production. Because one-carbon metabolism supplies the universal methyl donor for multiple methylation reactions, disrupting this pathway may broadly impair the adaptive methylation programs that support cancer cell plasticity (Islam et al., 2022). Among metabolic interventions, methionine restriction has attracted considerable attention. Methionine restriction reduces methylation potential by lowering intracellular SAM levels and altering the SAM/SAH ratio, thereby limiting methyltransferase activity. Dietary methionine restriction has been shown to alter one-carbon metabolism, suppress tumor growth in selected models, and modify responses to chemotherapy and radiotherapy (Gao et al., 2019). Pharmacological inhibition of methionine adenosyltransferase 2 A (MAT2A) represents a more genetically defined approach to targeting SAM metabolism. Its clearest therapeutic rationale has emerged in methylthioadenosine phosphorylase (MTAP)-deleted cancers, in which loss of MTAP creates a selective dependency on protein arginine methyltransferase 5 (PRMT5)-associated methylation and renders tumor cells particularly vulnerable to reductions in MAT2A-dependent SAM synthesis (Kalev et al., 2021). This synthetic-lethal framework has now progressed into early clinical evaluation. In a first-in-human phase I study, the oral MAT2A inhibitor AG-270/S095033 was administered to 40 patients with advanced malignancies enriched for CDKN2A/MTAP loss. Treatment produced maximal plasma SAM reductions of 54%–70% and decreased tumor symmetric dimethylarginine, providing pharmacodynamic evidence of MAT2A–PRMT5 pathway suppression. Two patients achieved partial responses and five additional patients maintained radiographically confirmed stable disease for at least 16 weeks, corresponding to a 16-week disease-control rate of 17.5% (Gounder et al., 2025). Although further development of AG-270/S095033 was subsequently paused in favor of newer MAT2A inhibitors, these data provide clinical proof-of-mechanism for SAM depletion in molecularly selected tumors and reinforce MTAP loss as the most established biomarker context for MAT2A-directed therapy. Representative clinical-stage compounds and available efficacy data are summarized in Table 2. In addition, inhibition of folate-dependent one-carbon enzymes such as methylenetetrahydrofolate dehydrogenase 2 (MTHFD2) disrupts one-carbon flux and further limits methyl donor production. These strategies preferentially target tumors with high dependence on one-carbon metabolism and adaptive epigenetic remodeling (Ren et al., 2024).

A potential advantage of targeting one-carbon metabolism is its capacity to influence multiple SAM-dependent methylation processes. However, the magnitude and direction of these effects are unlikely to be uniform because individual methyltransferases differ in their metabolic sensitivity and regulatory context. Metabolic interventions may therefore reshape several methylation layers simultaneously but to different extents, providing a rationale for combining metabolic interventions with selected DNMT-, EZH2-, or METTL3-directed therapies. By restricting methyl donor availability, interventions such as methionine restriction, MAT2A inhibition, and MTHFD2 inhibition may alter methylation-dependent adaptive programs and potentially enhance therapeutic responses in metabolically selected tumors.

However, the therapeutic potential of methionine restriction must be balanced against its systemic effects on the host. Methionine is required not only by tumor cells but also by normal tissues, and disruption of host methionine-cycle metabolism may have adverse consequences in patients with cancer-associated cachexia. Notably, the disruption of the methionine cycle in skeletal muscle promotes DNA hypomethylation, REDD1 induction, and muscle atrophy, whereas methionine supplementation restored DNMT3A-dependent DNA methylation and alleviated muscle wasting in experimental models (Lin et al., 2025). These findings highlight a potential host–tumor trade-off: although restricting methionine availability may expose metabolic vulnerabilities in tumor cells, excessive or prolonged restriction could aggravate muscle loss and nutritional deterioration in susceptible patients. Indeed, an early phase I dietary methionine-restriction study in patients with metastatic cancer demonstrated substantial reductions in circulating methionine but also reported gradual weight loss during prolonged dietary intervention (Epner et al., 2002). Therefore, the clinical translation of methionine restriction will require careful consideration of nutritional status, sarcopenia, treatment duration, tolerability, and patient compliance, particularly in cachectic populations.

The clinical development, representative efficacy, and major safety limitations of methylation- and one-carbon metabolism-targeted therapeutic strategies are summarized in Table 2, highlighting the marked differences in translational maturity across established DNA hypomethylating agents, histone methyltransferase inhibitors, emerging RNA methylation inhibitors, and metabolism-directed approaches.

### 5.5 Natural compounds and natural-product derivatives targeting the epigenetic–metabolic axis

Natural compounds occupy a distinctive position within the therapeutic landscape of methylation regulation. Unlike highly selective DNMT, EZH2, METTL3, or MAT2A inhibitors, most natural products do not act through a single epigenetic target. Instead, they can simultaneously alter metabolic signaling, redox state, methylation-enzyme abundance or activity, and the availability of cofactors required for methylation turnover. This pleiotropy has often been viewed as a pharmacological limitation; however, in the context of cancer plasticity, it may also provide a means of perturbing several components of the epigenetic–metabolic axis simultaneously. The critical mechanistic question is therefore not whether a natural compound globally increases or decreases methylation, but whether it reproducibly shifts a defined writer–eraser balance, metabolic dependency, or locus-specific methylation state in a manner that constrains malignant growth or adaptive cell-state transitions. Recent experimental studies increasingly support this more mechanistically resolved interpretation.

At the level of DNA methylation, recent studies indicate that structurally distinct dietary polyphenols can converge on the DNMT machinery while producing markedly different locus-specific outcomes. EGCG suppresses DNMT activity and DNMT3A/3B abundance in head and neck squamous cell carcinoma, reduces global DNA hypermethylation, restores silenced tumor-suppressor expression, and markedly inhibits xenograft growth, providing direct linkage between DNA-methylation remodeling and tumor suppression (Agarwal et al., 2023). A separate study in breast cancer cells showed that EGCG reduced 5 mC and IFI16-promoter methylation while restoring IFI16-associated innate immune signaling (Khan et al., 2022). Luteolin similarly inhibits DNMT and histone-methyltransferase activity, reduces promoter methylation of multiple tumor-suppressor genes, and restores their expression while suppressing colony formation and migration (Pramodh et al., 2022). Conversely, quercetin and resveratrol illustrate that anticancer methylation remodeling need not be uniformly demethylating: quercetin increased IL15-promoter methylation while inhibiting cancer-cell proliferation (Zhang M. et al., 2023), whereas resveratrol increased methylation at GLI2 and WNT4 regulatory elements, accompanied by increased H3K27me3, reduced histone acetylation, and suppression of Hedgehog/Wnt signaling in breast cancer cells (Kurzava Kendall et al., 2024). Together, these findings argue against a simplistic “natural compound–induced demethylation” model and instead support locus-selective redistribution of DNA methylation toward transcriptional states that are less permissive for malignant growth.

The same principle extends to histone methylation, where natural compounds can act on either the writer or the eraser arm and, importantly, can couple these epigenetic effects to metabolic signaling. In breast cancer cells, combined sulforaphane, genistein, and sodium butyrate treatment produced stronger suppression of DNMT3A/3B, EZH2, and SUV39H1 than the individual compounds, together with reductions in H3K27 and H3K9 methylation and enhanced growth inhibition (Sharma and Tollefsbol, 2022). Curcumin likewise decreases global lysine methylation in colorectal cancer cells and suppresses several methyltransferases, including EZH2, G9a, and MLL1, with genotype-dependent effects on apoptosis and ferroptosis (Santarelli et al., 2025). Other plant-derived compounds converge on EZH2 through distinct upstream mechanisms: corynoxine suppresses ESCC growth through an EZH2–DUSP5–ERK1/2 axis *in vitro* and in xenografts (Huang G. et al., 2024), while ailanthone suppresses EZH2-associated transcriptional programs and proliferation in renal-cell carcinoma (Zhu et al., 2024). These observations suggest that natural products can perturb repressive histone-methylation states through multiple entry points rather than behaving as conventional active-site methyltransferase inhibitors.

Particularly relevant to the metabolic–epigenetic axis are natural compounds that influence histone demethylation through upstream metabolic signaling. Pterostilbene provides one of the clearest recent examples. In experimental hepatocellular carcinoma, pterostilbene restored expression of genes involved in folate, methionine, and sarcosine metabolism while reducing promoter-associated H3K27me3. Mechanistically, AMPK inhibition abolished these transcriptional and epigenetic effects and reduced recruitment of the H3K27me3 demethylase KDM6A to the promoters of ALDH1L1, BHMT, and DMGDH (Boycott et al., 2025). This establishes a bidirectional circuit in which a natural stilbenoid activates metabolic signaling, modifies demethylase-associated chromatin states, and restores one-carbon metabolic programs. Such feedback is conceptually important because it moves natural products beyond the role of conventional epigenetic inhibitors: they can alter the metabolic state that determines how methylation enzymes operate, while methylation remodeling in turn reprograms metabolic gene expression.

Natural modulation of methylation can also occur through the cofactor requirements of eraser enzymes. Vitamin C is mechanistically distinct from polyphenols that alter writer abundance because it enhances Fe^2+^/α-KG-dependent TET activity. Recent work in AML demonstrated that pharmacological restoration of residual TET2 activity with vitamin C increased oxidized methylcytosine formation, promoted differentiation, reduced leukemic progression, and created a synthetic vulnerability to PARP inhibition. The combination of vitamin C and PARP inhibitors strongly suppressed self-renewal in murine and human AML models through accumulation of oxidized methylcytosines, chromatin-bound PARP1, replication stress, and differentiation (Brabson et al., 2023). This study provides a direct therapeutic extension of the writer–eraser framework developed above: the epigenetic–metabolic axis can be manipulated not only by inhibiting methyltransferases but also by restoring the metabolic cofactor environment required for active demethylation.

A rapidly expanding body of experimental work further places natural products within the m^6^A writer–eraser axis. Sulforaphene suppresses proliferation and promotes apoptosis in endometrioid ovarian cancer cells in association with METTL3 inhibition, and METTL3 gain- and loss-of-function experiments support a functional contribution of the writer to this response (Yu et al., 2023). Triptolide similarly suppresses oral squamous-cell carcinoma by disrupting the METTL3–m^6^A–IGF2BP2–SLC7A11 pathway, thereby linking a natural diterpenoid to m^6^A-dependent control of a ferroptosis-related metabolic gene (Xu et al., 2021). In a metabolically distinct setting, baicalin suppresses high-glucose/T2D-associated liver tumor progression by reducing METTL3-dependent m^6^A modification of HKDC1 and altering downstream JAK2/STAT1 signaling (Jiang et al., 2022). Curcumin has also been shown to inhibit prostate-cancer proliferation and migration through a METTL3/YTHDF2-regulated m^6^A modification of circ0030568 (Sun et al., 2024). Collectively, these data suggest that natural compounds can alter the writer side of the epitranscriptomic landscape at multiple levels, from METTL3 abundance to target-specific m^6^A deposition and reader-dependent RNA fate, thereby coupling RNA methylation to growth, stemness, migration, metabolic adaptation, and cell-death susceptibility.

The eraser arm of m^6^A regulation is equally tractable. Phenethyl isothiocyanate (PEITC) suppresses migration and invasion of non-small-cell lung cancer cells by reducing FTO, increasing m^6^A abundance, and destabilizing the FTO-regulated TLE1 program; FTO knockdown and overexpression experiments further demonstrated that FTO contributes functionally to the antimetastatic response, including *in vivo* tumor-growth assays (Zhang Q. C. et al., 2024). Rhein likewise targets FTO in multidrug-resistant AML, suppressing proliferation and migration, promoting apoptosis, inhibiting AKT/mTOR signaling, reducing tumor growth, and enhancing the activity of azacitidine (Zhang S. et al., 2024). These findings are particularly relevant to cancer plasticity because FTO-dependent RNA demethylation can support survival and drug-adaptive states, suggesting that naturally derived eraser modulators may be useful not simply as cytotoxic agents but as sensitizers that destabilize epitranscriptomic programs underlying therapeutic persistence.

Natural-product-derived analogues offer a further opportunity to convert broadly active scaffolds into more mechanistically tractable epigenetic agents. A recent example is EF24, a synthetic curcumin analogue that suppresses glioma growth and metastasis while inducing ferroptosis. Mechanistically, EF24 downregulates METTL3 and disrupts a METTL3–YTHDF1–NRF2–GPX4 epitranscriptomic circuit, thereby coupling m^6^A regulation directly to antioxidant metabolism and ferroptotic vulnerability (Yang et al., 2025). Together with pterostilbene and other optimized natural-product scaffolds, such studies illustrate a transition from descriptive phytochemistry toward mechanism-guided drug development, in which structural optimization is used to improve exposure and target engagement while retaining a defined epigenetic–metabolic mechanism.

Taken together, recent experimental evidence indicates that natural compounds are mechanistically heterogeneous modulators of the epigenetic–metabolic axis rather than a uniform class of “epigenetic drugs.” By acting on DNA and histone methylation writers, methylation erasers, and m^6^A regulatory machinery, these compounds can reshape context-dependent methylation states and thereby influence cancer growth and plasticity. Their biological effects, however, cannot be attributed to methylation alone, because many natural products simultaneously affect kinase signaling, redox homeostasis, mitochondrial function, inflammation, and cell-death pathways. Establishing epigenetic causality therefore requires direct interrogation of the relevant writer or eraser, locus- or transcript-specific methylation changes, and, ideally, genetic or pharmacological rescue. From a translational perspective, limited bioavailability, variable pharmacokinetics, incomplete target selectivity, and achievable intratumoral exposure remain major challenges. Accordingly, the greatest therapeutic potential of natural compounds may lie in mechanism-guided combination strategies that exploit metabolically defined vulnerabilities and reshape adaptive methylation states, as illustrated by vitamin C–PARP inhibition and rhein–azacitidine combinations.

### 5.6 Safety considerations and unintended consequences of methylation-targeted therapies

The therapeutic manipulation of methylation must be balanced against systemic toxicity and the physiological functions of epigenetic regulation in normal tissues. Hypomethylating agents such as decitabine are associated with clinically relevant hematologic toxicities, particularly neutropenia, thrombocytopenia, and anemia (Kantarjian et al., 2006). The safety profile of EZH2 inhibition requires careful interpretation, particularly given the increased incidence of hematologic second primary malignancies observed with tazemetostat, which prompted voluntary withdrawal from the U.S. market in 2026. Metabolic interventions introduce an additional layer of risk because they alter nutrient availability in both malignant and normal tissues. As discussed above, prolonged methionine restriction may be particularly problematic in patients with malnutrition, sarcopenia, or cancer cachexia (Lin et al., 2025). For emerging m^6^A-targeted therapies, including METTL3 inhibition, clinical safety data remain comparatively limited and require continued evaluation as these agents advance through clinical development.

A further concern is that disrupting methylation may itself reshape cancer cell plasticity in unintended ways. Because epigenetic states can constrain the range of transcriptional programs accessible to cancer cells, broadly perturbing methylation may suppress one resistant state while creating permissive conditions for alternative cell states. Notably, recent experimental evidence in prostate cancer showed that genetic or pharmacological suppression of EZH2 altered chromatin states and increased the diversity of lineage variants during tumor progression (Jacobi et al., 2026). Similarly, PRC2/EZH2 inhibition has been reported to induce diverse cell-state programs, including stem-like, EMT-associated, and developmental programs, accompanied by increased invasion, metastasis, and resistance in preclinical prostate cancer models (Yang et al., 2026). These observations suggest that epigenetic therapies may alter the repertoire of phenotypes available to surviving tumor cells rather than simply reversing a single malignant state. This possibility underscores the need for context-specific targeting, biomarker-guided patient selection, and longitudinal monitoring of cell-state changes during treatment. From the perspective of the adaptive methylation landscape, therapeutic efficacy should therefore be evaluated not only by immediate tumor response but also by whether treatment constrains or inadvertently expands the adaptive states accessible to residual cancer cells.

## 6 Mapping adaptive methylation landscapes

The concept of adaptive methylation landscapes implies that cancer cell plasticity can be associated with and functionally influenced by dynamic and heterogeneous epigenetic remodeling that cannot be fully captured by conventional bulk sequencing. Recent technological advances now enable visualization of methylation dynamics across individual cells, spatial niches, and metabolic contexts, providing new opportunities to investigate how metabolism is associated with and may influence cancer cell plasticity (Liu et al., 2025). Crucially, mapping an adaptive methylation landscape requires more than generating a static methylome. It requires linking cell-state-resolved methylation patterns to defined metabolic or environmental conditions and determining whether these patterns change reproducibly as the relevant pressure is introduced, removed, or altered. Accordingly, “mapping” in this context refers to resolving methylation states together with the metabolic condition, cellular phenotype, spatial location, and temporal or perturbational context in which those states occur.

Operationally, adaptive methylation landscapes could be measured by integrating cell-state-resolved methylation profiling with transcriptional and metabolic measurements. Single-cell or single-nucleus DNA methylome profiling can quantify methylation heterogeneity across individual cells and cellular states, while multimodal approaches such as scNMT-seq can jointly resolve DNA methylation, chromatin accessibility, and transcription within the same cells (Clark et al., 2018). These epigenetic measurements can be integrated with transcriptomic profiling to define phenotypic states and with metabolomic measurements to quantify relevant metabolic variables, including SAM/SAH balance and, where appropriate, metabolites or cofactors influencing demethylase activity. Parallel assessment of writer and eraser activity or expression can further help distinguish whether a methylation shift reflects altered methyl-group deposition, altered demethylation, or both. Most importantly, longitudinal sampling and controlled perturbation–recovery experiments will be required to determine whether methylation states shift reproducibly in response to metabolic or therapeutic pressure and whether these changes are reversible when the pressure is removed. Together, these measurements would enable adaptive methylation landscapes to be characterized in terms of their cellular heterogeneity, metabolic context, spatial organization, temporal dynamics, and association with phenotypic-state transitions.

Among these advances, single-cell multiomic technologies increasingly enable integrated profiling of DNA methylation, chromatin accessibility, transcriptomes, and, in selected platforms, histone modifications. Single-cell approaches resolve rare transitional populations, including drug-tolerant persister cells, stem-like intermediates, dormant cells, and lineage-switched cells, and reconstruct cell-state trajectories during tumor progression and therapeutic response (Liu F. et al., 2023; Chen et al., 2025). Spatial epigenomics complements these approaches by preserving tissue architecture while mapping molecular regulation within the tumor microenvironment. Integration of spatial transcriptomics with epigenetic profiling reveals how hypoxia, nutrient limitation, immune infiltration, and stromal interactions are associated with local methylation states and cellular heterogeneity (Koch, 2023; Zhang D. et al., 2023). An important dimension of adaptive methylation landscapes is their spatial and temporal heterogeneity. Within a single tumor, distinct anatomical regions, such as the hypoxic tumor core, invasive margin, and metastatic niche, are exposed to different levels of oxygen, nutrients, metabolites, stromal signals, and immune pressure, establishing distinct microenvironmental conditions that may favor region-specific epigenetic and cellular states (Lee et al., 2024). These landscapes may also evolve over time as tumors progress and encounter therapeutic pressure. Longitudinal epigenomic analyses of paired primary and recurrent gliomas have demonstrated substantial changes in DNA methylation during disease progression and treatment, with recent studies further linking methylation evolution to altered differentiation and stem-like cell states (Malta et al., 2024; Nomura et al., 2026). Longitudinal profiling before, during, and after treatment may therefore help distinguish transient stress responses from more persistent adaptive states. Integrating spatially resolved epigenomic profiling with longitudinal sampling and metabolic measurements will be essential for reconstructing how adaptive methylation landscapes evolve across anatomical space and disease time.

Functional interrogation has also been accelerated by CRISPR-based epigenome editing, which enables locus-specific manipulation of DNA methylation and chromatin states without altering genomic sequences. Combined with pooled screening, these tools facilitate identification of causal epigenetic regulators of EMP, stemness, dormancy, lineage switching, and therapy resistance (Fadul et al., 2023). In parallel, machine-learning and AI-based approaches are increasingly being applied to the integration and interpretation of epigenomic and multiomic datasets, including dimensionality reduction, cell-state classification, feature selection, and pattern discovery (Arslan et al., 2021). Despite their potential, these emerging approaches have important technical and analytical limitations. Single-cell DNA methylation sequencing remains constrained by sparse CpG coverage at the individual-cell level, which complicates robust reconstruction of cell-specific methylation states and often requires signal aggregation or computational approaches to address missing measurements (Tang et al., 2026). Apparent cell-to-cell differences should therefore not automatically be interpreted as biological heterogeneity without careful consideration of coverage depth, missingness, and computational imputation. Spatial epigenomic approaches similarly involve trade-offs among spatial resolution, genomic coverage, throughput, and technical complexity, which currently limit their scalability and broad application to clinical specimens (Crespo-Garcia and Esteller, 2026). These limitations are particularly relevant when attempting to resolve small or rare adaptive populations within heterogeneous tumor niches. CRISPR-based epigenome editing provides a powerful means of testing locus-specific causality, but editing efficiency and persistence can vary across genomic contexts, while off-target epigenetic modifications remain an important concern requiring careful validation (Galonska et al., 2018; McCutcheon et al., 2024). Similarly, AI-based integration of epigenomic and multi-omic datasets is challenged by the high dimensionality and relatively small sample sizes characteristic of epigenomic studies, as well as class imbalance and the need for rigorous independent validation to ensure model generalizability (Arslan et al., 2021). Predictive performance within a discovery cohort should therefore not be equated with biological causality or clinical utility without external validation and mechanistic confirmation. Accordingly, these technologies should be viewed as complementary and still-evolving tools rather than as established solutions for resolving adaptive methylation landscapes.

The greatest potential of these technologies lies in their integration. Combining single-cell multi-omics, spatial epigenomics, metabolomics, functional epigenome editing, and AI will enable progressively more resolved maps of adaptive methylation landscapes across space and time. Such multidimensional atlases could ultimately support patient stratification and inform therapeutic selection by stratifying patients according to dynamic epigenetic states, thereby guiding metabolism-directed therapies, epigenetic interventions, and rational combination strategies.

A central testable prediction of this framework is that metabolic perturbations that modify methyl-donor availability or demethylase cofactor conditions will not produce uniform methylation changes across all cancer cells. Instead, they should reproducibly redistribute cells among distinct methylation-associated states, with the magnitude and direction of the response depending on the pre-existing cell state, the balance between methylation writers and erasers, and the methylation machinery engaged. If the adaptive methylation landscape is functionally involved in plasticity, restoration of the metabolic condition should permit at least partial reversal of these methylation and cell-state distributions, whereas targeted disruption of the responsible methylation machinery should alter or prevent the corresponding state transition. This state-selective and potentially reversible redistribution distinguishes the adaptive methylation landscape framework from a general model in which metabolism merely produces global or static methylation changes.

## 7 Conclusion and perspectives

Cancer cell plasticity has long been viewed primarily as a consequence of genetic evolution. However, accumulating evidence indicates that the remarkable reversibility and phenotypic flexibility of cancer cells cannot be explained by genetic alterations alone. Instead, metabolic adaptation and epigenetic remodeling can jointly influence cellular identity in response to environmental and therapeutic pressures.

In this review, we highlight one-carbon metabolism as a major metabolic interface linking nutrient availability to DNA, histone, and RNA methylation. These methylation systems represent distinct but metabolically connected regulatory layers that can contribute to EMP, stemness, dormancy, lineage switching, immune evasion, and therapy resistance. Based on these observations, we propose the concept of the adaptive methylation landscape, a dynamic, spatially heterogeneous, and temporally evolving epigenetic architecture that can be remodeled in response to metabolic rewiring and microenvironmental pressures. This framework positions methylation as a metabolically responsive system that interprets environmental cues and may translate metabolic and environmental cues into changes in the range of phenotypic states accessible to cancer cells. We propose this concept as a testable framework for investigating how metabolic and methylation remodeling may contribute to the balance between phenotypic stability and adaptability during tumor evolution. This perspective also has important therapeutic implications. Future therapeutic strategies may be more effective if they disrupt the metabolic-methylation axis that sustains adaptive plasticity. Integrating metabolic intervention with epigenetic therapies, including mechanism-guided natural compounds or natural-product-derived agents where appropriate, together with emerging technologies such as single-cell multi-omics, spatial epigenomics, and metabolomics, may enable dynamic characterization and therapeutic manipulation of adaptive methylation landscapes. Importantly, therapeutic disruption of methylation must be approached with caution, as systemic toxicity and the potential induction or expansion of alternative plastic states may limit the benefits of broadly acting epigenetic or metabolic interventions.

Several limitations of the current review and proposed framework should be acknowledged. First, this is a narrative, non-systematic review, and the evidence discussed was selected to illustrate emerging mechanistic connections among one-carbon metabolism, methylation regulation, and cancer cell plasticity rather than to provide a comprehensive or quantitative synthesis. Second, much of the mechanistic evidence linking metabolic state to dynamic methylation remodeling and phenotypic plasticity is derived from cancer cell lines, organoids, and animal models, and its relevance across human tumors requires further validation. Experimental nutrient conditions may also differ substantially from the physiological metabolic environment, which should be considered when interpreting nutrient-dependent effects on one-carbon metabolism and methylation, as discussed in Section 2.1. Finally, the adaptive methylation landscape proposed here should be regarded as a conceptual and testable framework rather than an established biological model. Further studies integrating metabolic measurements with spatial and longitudinal epigenomic profiling will be required to determine its generalizability and clinical relevance.

Taken together, understanding how metabolic state shapes dynamic methylation programs may provide a useful framework for defining, monitoring, and therapeutically constraining the range of adaptive states available to cancer cells. Further characterization and therapeutic targeting of these adaptive methylation landscapes may ultimately provide new opportunities to understand tumor evolution and potentially improve the durability of precision cancer therapies.

## References

[B1] Agarwal A. Kansal V. Farooqi H. Prasad R. Singh V. K. (2023). Epigallocatechin gallate (EGCG), an active phenolic compound of green tea, inhibits tumor growth of head and neck cancer cells by targeting DNA hypermethylation. Biomedicines 11, 789. 10.3390/biomedicines11030789 36979768 PMC10045148

[B2] Akhmetkaliyev A. Alibrahim N. Shafiee D. Tulchinsky E. (2023). EMT/MET plasticity in cancer and go-or-grow decisions in quiescence: the two sides of the same coin? Mol. Cancer 22, 90. 10.1186/s12943-023-01793-z 37259089 PMC10230810

[B3] An Y. Duan H. (2022). The role of m6A RNA methylation in cancer metabolism. Mol. Cancer 21, 14. 10.1186/s12943-022-01500-4 35022030 PMC8753874

[B4] Arslan E. Schulz J. Rai K. (2021). Machine learning in epigenomics: insights into cancer biology and medicine. Biochim. Biophys. Acta Rev. Cancer 1876, 188588. 10.1016/j.bbcan.2021.188588 34245839 PMC8595561

[B5] Artika I. M. Arianti R. Demeny M. A. Kristof E. (2025). RNA modifications and their role in gene expression. Front. Mol. Biosci. 12, 1537861. 10.3389/fmolb.2025.1537861 40351534 PMC12061695

[B6] Atlasi Y. Stunnenberg H. G. (2017). The interplay of epigenetic marks during stem cell differentiation and development. Nat. Rev. Genet. 18, 643–658. 10.1038/nrg.2017.57 28804139

[B7] Banerjee A. Vathiotis I. Halder D. Sen U. Wang X. Shields M. D. (2025). Molecular landscape and therapeutic strategies of lung cancer lineage plasticity. J. Thorac. Oncol. 20, 1582–1593. 10.1016/j.jtho.2025.06.012 40751717

[B8] Bell R. E. Golan T. Sheinboim D. Malcov H. Amar D. Salamon A. (2016). Enhancer methylation dynamics contribute to cancer plasticity and patient mortality. Genome Res. 26, 601–611. 10.1101/gr.197194.115 26907635 PMC4864467

[B9] Bergin C. J. Zouggar A. Haebe J. R. Masibag A. N. Desrochers F. M. Reilley S. Y. (2021). G9a controls pluripotent-like identity and tumor-initiating function in human colorectal cancer. Oncogene 40, 1191–1202. 10.1038/s41388-020-01591-7 PMC7878189 33323965

[B10] Bian Y. Li W. Kremer D. M. Sajjakulnukit P. Li S. Crespo J. (2020). Cancer SLC43A2 alters T cell methionine metabolism and histone methylation. Nature 585, 277–282. 10.1038/s41586-020-2682-1 32879489 PMC7486248

[B11] Bian Y. Li W. Kremer D. M. Sajjakulnukit P. Li S. Crespo J. (2026). Author correction: cancer SLC43A2 alters T cell methionine metabolism and histone methylation. Nature 649, E3. 10.1038/s41586-025-09845-8 41388204

[B12] Blee A. M. Huang H. (2019). Lineage plasticity-mediated therapy resistance in prostate cancer. Asian J. Androl. 21, 241–248. 10.4103/aja.aja_41_18 29900883 PMC6498731

[B13] Boulias K. Greer E. L. (2023). Biological roles of adenine methylation in RNA. Nat. Rev. Genet. 24, 143–160. 10.1038/s41576-022-00534-0 36261710 PMC9974562

[B14] Boycott C. Beetch M. Lubecka-Gajewska K. Ramsey B. S. Torregrosa-Allen S. Elzey B. D. (2025). AMPK-Dependent epigenetic regulation of metabolism mediates the anti-cancer action of pterostilbene in hepatocellular carcinoma. Mol. Nutr. Food Res. 69, e70217. 10.1002/mnfr.70217 40888265 PMC12666732

[B15] Brabson J. P. Leesang T. Yap Y. S. Wang J. Lam M. Q. Fang B. (2023). Oxidized mC modulates synthetic lethality to PARP inhibitors for the treatment of leukemia. Cell Rep. 42, 112027. 10.1016/j.celrep.2023.112027 36848231 PMC9989506

[B16] Burr M. L. Sparbier C. E. Chan K. L. Chan Y. C. Kersbergen A. Lam E. Y. N. (2019). An evolutionarily conserved function of polycomb silences the MHC class I antigen presentation pathway and enables immune evasion in cancer. Cancer Cell 36, 385–401 e388. 10.1016/j.ccell.2019.08.008 31564637 PMC6876280

[B17] Cardenas H. Vieth E. Lee J. Segar M. Liu Y. Nephew K. P. (2014). TGF-Beta induces global changes in DNA methylation during the epithelial-to-mesenchymal transition in ovarian cancer cells. Epigenetics 9, 1461–1472. 10.4161/15592294.2014.971608 25470663 PMC4622747

[B18] Chakraborty A. A. Laukka T. Myllykoski M. Ringel A. E. Booker M. A. Tolstorukov M. Y. (2019). Histone demethylase KDM6A directly senses oxygen to control chromatin and cell fate. Science 363, 1217–1222. 10.1126/science.aaw1026 30872525 PMC7336390

[B19] Chen M. Fu R. Chen Y. Li L. Wang S. W. (2025). High-resolution, noninvasive single-cell lineage tracing in mice and humans based on DNA methylation epimutations. Nat. Methods 22, 488–498. 10.1038/s41592-024-02567-1 39820752

[B20] Clark S. J. Argelaguet R. Kapourani C. A. Stubbs T. M. Lee H. J. Alda-Catalinas C. (2018). scNMT-seq enables joint profiling of chromatin accessibility DNA methylation and transcription in single cells. Nat. Commun. 9, 781. 10.1038/s41467-018-03149-4 29472610 PMC5823944

[B21] Crespo-Garcia E. Esteller M. (2026). Spatial biology in cancer epigenetics. Mol. Oncol. 20, 2393–2409. 10.1002/1878-0261.70310 42487511 PMC13394033

[B22] Dai Z. Mentch S. J. Gao X. Nichenametla S. N. Locasale J. W. (2018). Methionine metabolism influences genomic architecture and gene expression through H3K4me3 peak width. Nat. Commun. 9, 1955. 10.1038/s41467-018-04426-y 29769529 PMC5955993

[B23] Dai Z. Ramesh V. Locasale J. W. (2020). The evolving metabolic landscape of chromatin biology and epigenetics. Nat. Rev. Genet. 21, 737–753. 10.1038/s41576-020-0270-8 32908249 PMC8059378

[B24] Davies A. Zoubeidi A. Beltran H. Selth L. A. (2023). The transcriptional and epigenetic landscape of cancer cell lineage plasticity. Cancer Discov. 13, 1771–1788. 10.1158/2159-8290.cd-23-0225 PMC10527883 37470668

[B25] Dekhne A. S. Hou Z. Gangjee A. Matherly L. H. (2020). Therapeutic targeting of mitochondrial one-carbon metabolism in cancer. Mol. Cancer Ther. 19, 2245–2255. 10.1158/1535-7163.MCT-20-0423 32879053 PMC7921203

[B26] Deng X. Qing Y. Horne D. Huang H. Chen J. (2023). The roles and implications of RNA m(6)A modification in cancer. Nat. Rev. Clin. Oncol. 20, 507–526. 10.1038/s41571-023-00774-x 37221357 PMC12466201

[B27] Dersh D. Phelan J. D. Gumina M. E. Wang B. Arbuckle J. H. Holly J. (2021). Genome-wide screens identify lineage- and tumor-specific genes modulating MHC-I- and MHC-II-restricted immunosurveillance of human lymphomas. Immunity 54, 116–131 e110. 10.1016/j.immuni.2020.11.002 33271120 PMC7874576

[B28] Dong C. Wu Y. Yao J. Wang Y. Yu Y. Rychahou P. G. (2012). G9a interacts with snail and is critical for Snail-mediated E-cadherin repression in human breast cancer. J. Clin. Invest. 122, 1469–1486. 10.1172/JCI57349 22406531 PMC3314447

[B29] Ducker G. S. Rabinowitz J. D. (2017). One-carbon metabolism in health and disease. Cell Metab. 25, 27–42. 10.1016/j.cmet.2016.08.009 27641100 PMC5353360

[B30] Epner D. E. Morrow S. Wilcox M. Houghton J. L. (2002). Nutrient intake and nutritional indexes in adults with metastatic cancer on a phase I clinical trial of dietary methionine restriction. Nutr. Cancer 42, 158–166. 10.1207/S15327914NC422_2 12416254

[B31] Esteller M. Dawson M. A. Kadoch C. Rassool F. V. Jones P. A. Baylin S. B. (2024). The epigenetic hallmarks of cancer. Cancer Discov. 14, 1783–1809. 10.1158/2159-8290.cd-24-0296 39363741

[B32] Etchegaray J. P. Mostoslavsky R. (2016). Interplay between metabolism and epigenetics: a nuclear adaptation to environmental changes. Mol. Cell 62, 695–711. 10.1016/j.molcel.2016.05.029 27259202 PMC4893201

[B33] Fadul S. M. Arshad A. Mehmood R. (2023). CRISPR-Based epigenome editing: mechanisms and applications. Epigenomics 15, 1137–1155. 10.2217/epi-2023-0281 37990877

[B34] Fenaux P. Mufti G. J. Hellstrom-Lindberg E. Santini V. Finelli C. Giagounidis A. (2009). Efficacy of azacitidine compared with that of conventional care regimens in the treatment of higher-risk myelodysplastic syndromes: a randomised, open-label, phase III study. Lancet Oncol. 10, 223–232. 10.1016/s1470-2045(09)70003-8 19230772 PMC4086808

[B35] Friso S. Udali S. De Santis D. Choi S. W. (2017). One-carbon metabolism and epigenetics. Mol. Asp. Med. 54, 28–36. 10.1016/j.mam.2016.11.007 27876555

[B36] Galle E. Thienpont B. Cappuyns S. Venken T. Busschaert P. Van Haele M. (2020). DNA methylation-driven EMT is a common mechanism of resistance to various therapeutic agents in cancer. Clin. Epigenetics 12, 27. 10.1186/s13148-020-0821-z 32059745 PMC7023776

[B37] Galonska C. Charlton J. Mattei A. L. Donaghey J. Clement K. Gu H. (2018). Genome-wide tracking of dCas9-methyltransferase footprints. Nat. Commun. 9, 597. 10.1038/s41467-017-02708-5 29426832 PMC5807365

[B38] Gan L. Xu M. Hua R. Tan C. Zhang J. Gong Y. (2018). The polycomb group protein EZH2 induces epithelial-mesenchymal transition and pluripotent phenotype of gastric cancer cells by binding to PTEN promoter. J. Hematol. Oncol. 11, 9. 10.1186/s13045-017-0547-3 29335012 PMC5769437

[B39] Gao X. Sanderson S. M. Dai Z. Reid M. A. Cooper D. E. Lu M. (2019). Dietary methionine influences therapy in mouse cancer models and alters human metabolism. Nature 572, 397–401. 10.1038/s41586-019-1437-3 31367041 PMC6951023

[B40] Gao Y. Siyu Z. Zhang X. Du Y. Ni T. Hao S. (2024). Crosstalk between metabolic and epigenetic modifications during cell carcinogenesis. iScience 27, 111359. 10.1016/j.isci.2024.111359 39660050 PMC11629229

[B41] Garbo S. Zwergel C. Battistelli C. (2021). m6A RNA methylation and beyond - the epigenetic machinery and potential treatment options. Drug Discov. Today 26, 2559–2574. 10.1016/j.drudis.2021.06.004 34126238

[B42] Ghasemi S. (2020). Cancer's epigenetic drugs: where are they in the cancer medicines? Pharmacogenomics J. 20, 367–379. 10.1038/s41397-019-0138-5 31819161

[B43] Gounder M. Schoffski P. Jones R. L. Agulnik M. Cote G. M. Villalobos V. M. (2020). Tazemetostat in advanced epithelioid sarcoma with loss of INI1/SMARCB1: an international, open-label, phase 2 basket study. Lancet Oncol. 21, 1423–1432. 10.1016/S1470-2045(20)30451-4 33035459

[B44] Gounder M. Johnson M. Heist R. S. Shapiro G. I. Postel-Vinay S. Wilson F. H. (2025). MAT2A inhibitor AG-270/S095033 in patients with advanced malignancies: a phase I trial. Nat. Commun. 16, 423. 10.1038/s41467-024-55316-5 39762248 PMC11704051

[B45] Greenberg M. V. C. Bourc'his D. (2019). The diverse roles of DNA methylation in Mammalian development and disease. Nat. Rev. Mol. Cell Biol. 20, 590–607. 10.1038/s41580-019-0159-6 31399642

[B46] Guan A. Wong J. J. (2024). Targeting RNA modifications with pharmacological agents: new frontiers in cancer therapy. Cancer Med. 13, e6989. 10.1002/cam4.6989 38545841 PMC10974741

[B47] Haerinck J. Goossens S. Berx G. (2023). The epithelial-mesenchymal plasticity landscape: principles of design and mechanisms of regulation. Nat. Rev. Genet. 24, 590–609. 10.1038/s41576-023-00601-0 37169858

[B48] Hanahan D. (2022). Hallmarks of cancer: new dimensions. Cancer Discov. 12, 31–46. 10.1158/2159-8290.cd-21-1059 35022204

[B49] He L. Li H. Wu A. Peng Y. Shu G. Yin G. (2019). Functions of N6-methyladenosine and its role in cancer. Mol. Cancer 18, 176. 10.1186/s12943-019-1109-9 31801551 PMC6892141

[B50] Hoffman R. M. (2019). Is the hoffman effect for methionine overuse analogous to the warburg effect for glucose overuse in cancer? Methods Mol. Biol. 1866, 273–278. 10.1007/978-1-4939-8796-2_21 30725423

[B51] Hua X. Xu Q. Wu R. Sun W. Gu Y. Zhu S. (2024). ALKBH5 promotes non-small cell lung cancer progression and susceptibility to anti-PD-L1 therapy by modulating interactions between tumor and macrophages. J. Exp. Clin. Cancer Res. 43, 164. 10.1186/s13046-024-03073-0 38872221 PMC11177518

[B52] Huang Q. Mo J. Liao Z. Chen X. Zhang B. (2022). The RNA m(6)A writer WTAP in diseases: structure, roles, and mechanisms. Cell Death Dis. 13, 852. 10.1038/s41419-022-05268-9 36207306 PMC9546849

[B53] Huang G. Xu J. Li Y. Song L. Wen C. Ruan Q. (2024a). Corynoxine exerts the anti-tumor effect on esophageal squamous cell carcinoma principally via the EZH2-DUSP5-ERK1/2-mediated cell growth inhibition. Phytomedicine 135, 156103. 10.1016/j.phymed.2024.156103 39383633

[B54] Huang W. Zhu Q. Shi Z. Tu Y. Li Q. Zheng W. (2024b). Dual inhibitors of DNMT and HDAC induce viral mimicry to induce antitumour immunity in breast cancer. Cell Death Discov. 10, 143. 10.1038/s41420-024-01895-7 PMC10943227 38490978

[B55] Huo M. Zhang J. Huang W. Wang Y. (2021). Interplay among metabolism, epigenetic modifications, and gene expression in cancer. Front. Cell Dev. Biol. 9, 793428. 10.3389/fcell.2021.793428 PMC8740611 35004688

[B56] Huo C. Wang Y. Li R. Xie X. Lyu W. Chen D. (2026). Lineage plasticity: a new dilemma in lung cancer treatment. Mol. Biol. Rep. 53, 362. 10.1007/s11033-026-11516-4 41636935

[B57] Islam A. Shaukat Z. Hussain R. Gregory S. L. (2022). One-carbon and polyamine metabolism as cancer therapy targets. Biomolecules 12, 1902. 10.3390/biom12121902 36551330 PMC9775183

[B58] Izzo L. T. Affronti H. C. Wellen K. E. (2021). The bidirectional relationship between cancer epigenetics and metabolism. Annu. Rev. Cancer Biol. 5, 235–257. 10.1146/annurev-cancerbio-070820-035832 34109280 PMC8186467

[B59] Jacobi J. J. Wadosky K. M. Jaiswal N. Zhang X. Wang Y. Singh P. K. (2026). EZH2 suppression diversifies prostate cancer lineage variant evolution and lacks efficacy in inhibiting disease progression. Cancer Res. 86, 889–908. 10.1158/0008-5472.can-25-2747 41223330 PMC12940094

[B60] Jia G. Fu Y. Zhao X. Dai Q. Zheng G. Yang Y. (2011). N6-methyladenosine in nuclear RNA is a major substrate of the obesity-associated FTO. Nat. Chem. Biol. 7, 885–887. 10.1038/nchembio.687 22002720 PMC3218240

[B61] Jiang X. Liu B. Nie Z. Duan L. Xiong Q. Jin Z. (2021). The role of m6A modification in the biological functions and diseases. Signal Transduct. Target Ther. 6, 74. 10.1038/s41392-020-00450-x PMC7897327 33611339

[B62] Jiang H. Yao Q. An Y. Fan L. Wang J. Li H. (2022). Baicalin suppresses the progression of type 2 diabetes-induced liver tumor through regulating METTL3/m(6)A/HKDC1 axis and downstream p-JAK2/STAT1/clevaged Capase3 pathway. Phytomedicine 94, 153823. 10.1016/j.phymed.2021.153823 34763315

[B63] Jiao C. Q. Hu C. Sun M. H. Li Y. Wu C. Xu F. (2025). Targeting METTL3 mitigates venetoclax resistance via proteasome-mediated modulation of MCL1 in acute myeloid leukemia. Cell Death Dis. 16, 233. 10.1038/s41419-025-07560-w 40169588 PMC11962166

[B64] Jin M. L. Jeong K. W. (2023). Histone modifications in drug-resistant cancers: from a cancer stem cell and immune evasion perspective. Exp. Mol. Med. 55, 1333–1347. 10.1038/s12276-023-01014-z 37394580 PMC10394043

[B65] Jones P. A. Ohtani H. Chakravarthy A. De Carvalho D. D. (2019). Epigenetic therapy in immune-oncology. Nat. Rev. Cancer 19, 151–161. 10.1038/s41568-019-0109-9 30723290

[B66] Kaelin W. G. Jr. Mcknight S. L. (2013). Influence of metabolism on epigenetics and disease. Cell 153, 56–69. 10.1016/j.cell.2013.03.004 23540690 PMC3775362

[B67] Kaiser P. (2020). Methionine dependence of cancer. Biomolecules 10, 568. 10.3390/biom10040568 32276408 PMC7226524

[B68] Kalev P. Hyer M. L. Gross S. Konteatis Z. Chen C. C. Fletcher M. (2021). MAT2A inhibition blocks the growth of MTAP-Deleted cancer cells by reducing PRMT5-dependent mRNA splicing and inducing DNA damage. Cancer Cell 39, 209–224 e211. 10.1016/j.ccell.2020.12.010 33450196

[B69] Kang N. Eccleston M. Clermont P. L. Latarani M. Male D. K. Wang Y. (2020). EZH2 inhibition: a promising strategy to prevent cancer immune editing. Epigenomics 12, 1457–1476. 10.2217/epi-2020-0186 32938196 PMC7607396

[B70] Kantarjian H. Issa J. P. Rosenfeld C. S. Bennett J. M. Albitar M. Dipersio J. (2006). Decitabine improves patient outcomes in myelodysplastic syndromes: results of a phase III randomized study. Cancer 106, 1794–1803. 10.1002/cncr.21792 16532500

[B71] Khalil B. D. Sanchez R. Rahman T. Rodriguez-Tirado C. Moritsch S. Martinez A. R. (2022). An NR2F1-specific agonist suppresses metastasis by inducing cancer cell dormancy. J. Exp. Med. 219, e20210836. 10.1084/jem.20210836 34812843 PMC8614154

[B72] Khan M. I. Nur S. M. Abdulaal W. H. (2022). A study on DNA methylation modifying natural compounds identified EGCG for induction of IFI16 gene expression related to the innate immune response in cancer cells. Oncol. Lett. 24, 218. 10.3892/ol.2022.13339 35707762 PMC9178671

[B73] Kim D. J. (2026). Molecular and immune mechanisms governing cancer metastasis, including dormancy, microenvironmental niches, and tumor-specific programs. Int. J. Mol. Sci. 27, 875. 10.3390/ijms27020875 41596524 PMC12840987

[B74] Koch L. (2023). Spatial epigenomics in single cells. Nat. Rev. Genet. 24, 1. 10.1038/s41576-022-00552-y 36348049

[B75] Ku S. Y. Rosario S. Wang Y. Mu P. Seshadri M. Goodrich Z. W. (2017). Rb1 and Trp53 cooperate to suppress prostate cancer lineage plasticity, metastasis, and antiandrogen resistance. Science 355, 78–83. 10.1126/science.aah4199 28059767 PMC5367887

[B76] Kurzava Kendall L. Ma Y. Yang T. Lubecka K. Stefanska B. (2024). Epigenetic effects of resveratrol on oncogenic signaling in breast cancer. Nutrients 16, 699. 10.3390/nu16050699 38474826 PMC10934115

[B77] Lan X. Field M. S. Stover P. J. (2018). Cell cycle regulation of folate-mediated one-carbon metabolism. Wiley Interdiscip. Rev. Syst. Biol. Med. 10, e1426. 10.1002/wsbm.1426 29889360 PMC11875019

[B78] Lee P. Chandel N. S. Simon M. C. (2020). Cellular adaptation to hypoxia through hypoxia inducible factors and beyond. Nat. Rev. Mol. Cell Biol. 21, 268–283. 10.1038/s41580-020-0227-y 32144406 PMC7222024

[B79] Lee S. Kim G. Lee J. Lee A. C. Kwon S. (2024). Mapping cancer biology in space: applications and perspectives on spatial omics for oncology. Mol. Cancer 23, 26. 10.1186/s12943-024-01941-z 38291400 PMC10826015

[B80] Lee H. Kim B. Park J. Park S. Yoo G. Yum S. (2025). Cancer stem cells: landscape, challenges and emerging therapeutic innovations. Signal Transduct. Target Ther. 10, 248. 10.1038/s41392-025-02360-2 40759634 PMC12322150

[B81] Lin K. Wei L. Wang R. Li L. Song S. Wang F. (2025). Disrupted methionine cycle triggers muscle atrophy in cancer cachexia through epigenetic regulation of REDD1. Cell Metab. 37, 460–476 e468. 10.1016/j.cmet.2024.10.017 39729999

[B82] Lio C. J. Yue X. Lopez-Moyado I. F. Tahiliani M. Aravind L. Rao A. (2020). TET methylcytosine oxidases: new insights from a decade of research. J. Biosci. 45, 21. 31965999 PMC7216820

[B83] Liu F. Wang Y. Gu H. Wang X. (2023a). Technologies and applications of single-cell DNA methylation sequencing. Theranostics 13, 2439–2454. 10.7150/thno.82582 PMC10196823 37215576

[B84] Liu J. Huang H. Zhang M. Qing G. Liu H. (2023b). Intertwined regulation between RNA m(6)A modification and cancer metabolism. Cell Insight 2, 100075. 10.1016/j.cellin.2022.100075 PMC10120304 37192910

[B85] Liu R. Zhao E. Yu H. Yuan C. Abbas M. N. Cui H. (2023c). Methylation across the central dogma in health and diseases: new therapeutic strategies. Signal Transduct. Target Ther. 8, 310. 10.1038/s41392-023-01528-y PMC10449936 37620312

[B86] Liu M. Yue Y. Chen X. Xian K. Dong C. Shi M. (2025). Genome-coverage single-cell histone modifications for embryo lineage tracing. Nature 640, 828–839. 10.1038/s41586-025-08656-1 40011786 PMC12003199

[B87] Lu C. Thompson C. B. (2012). Metabolic regulation of epigenetics. Cell Metab. 16, 9–17. 10.1016/j.cmet.2012.06.001 22768835 PMC3392647

[B88] Lv J. Wu Q. Li K. Bai K. Yu H. Zhuang J. (2023). Lysine N-methyltransferase SETD7 promotes bladder cancer progression and immune escape via STAT3/PD-L1 Cascade. Int. J. Biol. Sci. 19, 3744–3761. 10.7150/ijbs.87182 37564199 PMC10411476

[B89] Lyko F. (2018). The DNA methyltransferase family: a versatile toolkit for epigenetic regulation. Nat. Rev. Genet. 19, 81–92. 10.1038/nrg.2017.80 29033456

[B90] Maddocks O. D. K. Athineos D. Cheung E. C. Lee P. Zhang T. Van Den Broek N. J. F. (2017). Modulating the therapeutic response of tumours to dietary serine and glycine starvation. Nature 544, 372–376. 10.1038/nature22056 28425994

[B91] Malta T. M. Sabedot T. S. Morosini N. S. Datta I. Garofano L. Vallentgoed W. (2024). The epigenetic evolution of glioma is determined by the IDH1 mutation status and treatment regimen. Cancer Res. 84, 741–756. 10.1158/0008-5472.can-23-2093 38117484 PMC10911804

[B92] Marine J. C. Dawson S. J. Dawson M. A. (2020). Non-genetic mechanisms of therapeutic resistance in cancer. Nat. Rev. Cancer 20, 743–756. 10.1038/s41568-020-00302-4 33033407

[B93] Mccutcheon S. R. Rohm D. Iglesias N. Gersbach C. A. (2024). Epigenome editing technologies for discovery and medicine. Nat. Biotechnol. 42, 1199–1217. 10.1038/s41587-024-02320-1 39075148

[B94] Meng F. Li J. Xia Z. Wang Q. Sun Q. Wang S. (2025). Persistent lineage plasticity driving lung cancer development and progression. Clin. Transl. Med. 15, e70458. 10.1002/ctm2.70458 40847555 PMC12373981

[B95] Mentch S. J. Locasale J. W. (2016). One-carbon metabolism and epigenetics: understanding the specificity. Ann. N. Y. Acad. Sci. 1363, 91–98. 10.1111/nyas.12956 26647078 PMC4801744

[B96] Mentch S. J. Mehrmohamadi M. Huang L. Liu X. Gupta D. Mattocks D. (2015). Histone methylation dynamics and gene regulation occur through the sensing of one-carbon metabolism. Cell Metab. 22, 861–873. 10.1016/j.cmet.2015.08.024 26411344 PMC4635069

[B97] Metzger E. Wissmann M. Yin N. Muller J. M. Schneider R. Peters A. H. (2005). LSD1 demethylates repressive histone marks to promote androgen-receptor-dependent transcription. Nature 437, 436–439. 10.1038/nature04020 16079795

[B98] Moroz-Omori E. V. Huang D. Kumar Bedi R. Cheriyamkunnel S. J. Bochenkova E. Dolbois A. (2021). METTL3 inhibitors for epitranscriptomic modulation of cellular processes. ChemMedChem 16, 3035–3043. 10.1002/cmdc.202100291 34237194 PMC8518639

[B99] Morschhauser F. Tilly H. Chaidos A. Mckay P. Phillips T. Assouline S. (2020). Tazemetostat for patients with relapsed or refractory follicular lymphoma: an open-label, single-arm, multicentre, phase 2 trial. Lancet Oncol. 21, 1433–1442. 10.1016/S1470-2045(20)30441-1 33035457 PMC8427481

[B100] Nachiyappan A. Gupta N. Taneja R. (2022). EHMT1/EHMT2 in EMT, cancer stemness and drug resistance: emerging evidence and mechanisms. FEBS J. 289, 1329–1351. 10.1111/febs.16334 34954891

[B101] Niederst M. J. Sequist L. V. Poirier J. T. Mermel C. H. Lockerman E. L. Garcia A. R. (2015). RB loss in resistant EGFR mutant lung adenocarcinomas that transform to small-cell lung cancer. Nat. Commun. 6, 6377. 10.1038/ncomms7377 25758528 PMC4357281

[B102] Nilsson R. Jain M. Madhusudhan N. Sheppard N. G. Strittmatter L. Kampf C. (2014). Metabolic enzyme expression highlights a key role for MTHFD2 and the mitochondrial folate pathway in cancer. Nat. Commun. 5, 3128. 10.1038/ncomms4128 24451681 PMC4106362

[B103] Nishiyama A. Nakanishi M. (2021). Navigating the DNA methylation landscape of cancer. Trends Genet. 37, 1012–1027. 10.1016/j.tig.2021.05.002 34120771

[B104] Nomura M. Raviram R. Schiffman J. S. Bussema L. Lu V. Wheeler N. (2026). Longitudinal changes in DNA methylation in IDH-Mutant glioma fuel disease progression through altered cell state differentiation. Nat. Genet. 58, 1651–1660. 10.1038/s41588-026-02642-7 42332268 PMC13364636

[B105] Padeken J. Methot S. P. Gasser S. M. (2022). Establishment of H3K9-methylated heterochromatin and its functions in tissue differentiation and maintenance. Nat. Rev. Mol. Cell Biol. 23, 623–640. 10.1038/s41580-022-00483-w 35562425 PMC9099300

[B106] Pan S. Fan M. Liu Z. Li X. Wang H. (2021). Serine, glycine and one-carbon metabolism in cancer. Int. J. Oncol. 58, 158–170. 10.3892/ijo.2020.5158 33491748 PMC7864012

[B107] Pandit M. Kil Y. S. Ahn J. H. Pokhrel R. H. Gu Y. Mishra S. (2023). Methionine consumption by cancer cells drives a progressive upregulation of PD-1 expression in CD4 T cells. Nat. Commun. 14, 2593. 10.1038/s41467-023-38316-9 37147330 PMC10162977

[B108] Paris J. Morgan M. Campos J. Spencer G. J. Shmakova A. Ivanova I. (2019). Targeting the RNA m(6)A reader YTHDF2 selectively compromises cancer stem cells in acute myeloid leukemia. Cell Stem Cell 25, 137–148 e136. 10.1016/j.stem.2019.03.021 31031138 PMC6617387

[B109] Pavlova N. N. Thompson C. B. (2016). The emerging hallmarks of cancer metabolism. Cell Metab. 23, 27–47. 10.1016/j.cmet.2015.12.006 PMC4715268 26771115

[B110] Perez-Gonzalez A. Bevant K. Blanpain C. (2023). Cancer cell plasticity during tumor progression, metastasis and response to therapy. Nat. Cancer 4, 1063–1082. 10.1038/s43018-023-00595-y 37537300 PMC7615147

[B111] Pistore C. Giannoni E. Colangelo T. Rizzo F. Magnani E. Muccillo L. (2017). DNA methylation variations are required for epithelial-to-mesenchymal transition induced by cancer-associated fibroblasts in prostate cancer cells. Oncogene 36, 5551–5566. 10.1038/onc.2017.159 28581528

[B112] Pitarresi J. R. Stanger B. Z. (2024). Cellular origins and lineage plasticity in cancer. Cold Spring Harb. Perspect. Med. 14, a041389. 10.1101/cshperspect.a041389 PMC11146313 37604590

[B113] Poulard C. Noureddine L. M. Pruvost L. Le Romancer M. (2021). Structure, activity, and function of the protein lysine methyltransferase G9a. Life (Basel) 11, 1082. 10.3390/life11101082 34685453 PMC8541646

[B114] Pramodh S. Raina R. Hussain A. Bagabir S. A. Haque S. Raza S. T. (2022). Luteolin causes 5'CpG demethylation of the promoters of TSGs and modulates the aberrant histone modifications, restoring the expression of TSGs in human cancer cells. Int. J. Mol. Sci. 23, 4067. 10.3390/ijms23074067 35409426 PMC8999529

[B115] Quintanal-Villalonga A. Chan J. M. Yu H. A. Pe'er D. Sawyers C. L. Sen T. (2020). Lineage plasticity in cancer: a shared pathway of therapeutic resistance. Nat. Rev. Clin. Oncol. 17, 360–371. 10.1038/s41571-020-0340-z 32152485 PMC7397755

[B116] Ren X. Wang X. Zheng G. Wang S. Wang Q. Yuan M. (2024). Targeting one-carbon metabolism for cancer immunotherapy. Clin. Transl. Med. 14, e1521. 10.1002/ctm2.1521 38279895 PMC10819114

[B117] Santarelli R. Curra P. Di Crosta M. Gonnella R. Gilardini Montani M. S. Cirone M. (2025). Changes in lysine methylation contribute to the cytotoxicity of curcumin in Colon cancer cells. Molecules 30, 335. 10.3390/molecules30020335 39860204 PMC11767838

[B118] Sharma M. Tollefsbol T. O. (2022). Combinatorial epigenetic mechanisms of sulforaphane, genistein and sodium butyrate in breast cancer inhibition. Exp. Cell Res. 416, 113160. 10.1016/j.yexcr.2022.113160 35447103 PMC12302910

[B119] Sharma S. V. Lee D. Y. Li B. Quinlan M. P. Takahashi F. Maheswaran S. (2010). A chromatin-mediated reversible drug-tolerant state in cancer cell subpopulations. Cell 141, 69–80. 10.1016/j.cell.2010.02.027 20371346 PMC2851638

[B120] Shen C. Sheng Y. Zhu A. C. Robinson S. Jiang X. Dong L. (2020). RNA demethylase ALKBH5 selectively promotes tumorigenesis and cancer stem cell self-renewal in acute myeloid leukemia. Cell Stem Cell 27, 64–80 e69. 10.1016/j.stem.2020.04.009 32402250 PMC7335338

[B121] Shi Y. Lan F. Matson C. Mulligan P. Whetstine J. R. Cole P. A. (2004). Histone demethylation mediated by the nuclear amine oxidase homolog LSD1. Cell 119, 941–953. 10.1016/j.cell.2004.12.012 15620353

[B122] Shi R. Liao C. Zhang Q. (2021). Hypoxia-driven effects in cancer: characterization, mechanisms, and therapeutic implications. Cells 10, 678. 10.3390/cells10030678 PMC8003323 33808542

[B123] Shi Z. D. Pang K. Wu Z. X. Dong Y. Hao L. Qin J. X. (2023). Tumor cell plasticity in targeted therapy-induced resistance: mechanisms and new strategies. Signal Transduct. Target Ther. 8, 113. 10.1038/s41392-023-01383-x 36906600 PMC10008648

[B124] Singh D. K. Carcamo S. Farias E. F. Hasson D. Zheng W. Sun D. (2023). 5-Azacytidine- and retinoic-acid-induced reprogramming of DCCs into dormancy suppresses metastasis via restored TGF-beta-SMAD4 signaling. Cell Rep. 42, 112560. 10.1016/j.celrep.2023.112560 37267946 PMC10592471

[B125] Sosa M. S. Parikh F. Maia A. G. Estrada Y. Bosch A. Bragado P. (2015). NR2F1 controls tumour cell dormancy via SOX9-and RARbeta-driven quiescence programmes. Nat. Commun. 6, 6170. 10.1038/ncomms7170 25636082 PMC4313575

[B126] Sun L. Zhang H. Gao P. (2022). Metabolic reprogramming and epigenetic modifications on the path to cancer. Protein Cell 13, 877–919. 10.1007/s13238-021-00846-7 34050894 PMC9243210

[B127] Sun W. Liu R. Gao X. Lin Z. Tang H. Cui H. (2023). Targeting serine-glycine-one-carbon metabolism as a vulnerability in cancers. Biomark. Res. 11, 48. 10.1186/s40364-023-00487-4 PMC10161514 37147729

[B128] Sun X. Huang X. Liu L. Shen W. Zheng F. Liu M. (2024). Anti-cancer role of curcumin in prostate cancer cells via regulation of m6A-modified circ0030568-FMR1 signaling pathway. Transl. Androl. Urol. 13, 2358–2375. 10.21037/tau-24-276 39698574 PMC11650353

[B129] Tang S. Li S. Zhang G. Lyu A. Li H. Chen S. (2026). Dissecting epigenetic heterogeneity in single-cell DNA methylomes with a unified framework. Nat. Commun. 17, 6469. 10.1038/s41467-026-73171-4 42140984 PMC13376737

[B130] Thienpont B. Steinbacher J. Zhao H. D'anna F. Kuchnio A. Ploumakis A. (2016). Tumour hypoxia causes DNA hypermethylation by reducing TET activity. Nature 537, 63–68. 10.1038/nature19081 27533040 PMC5133388

[B131] Turna S. Demokan S. (2026). Epigenetic alterations in cancer metastasis: molecular mechanisms and implications for precision oncology. Front. Oncol. 16, 1788808. 10.3389/fonc.2026.1788808 42137145 PMC13167426

[B132] Vande Voorde J. Ackermann T. Pfetzer N. Sumpton D. Mackay G. Kalna G. (2019). Improving the metabolic fidelity of cancer models with a physiological cell culture medium. Sci. Adv. 5, eaau7314. 10.1126/sciadv.aau7314 30613774 PMC6314821

[B133] Vander Heiden M. G. Cantley L. C. Thompson C. B. (2009). Understanding the warburg effect: the metabolic requirements of cell proliferation. Science 324, 1029–1033. 10.1126/science.1160809 19460998 PMC2849637

[B134] Villa E. Sahu U. O'hara B. P. Ali E. S. Helmin K. A. Asara J. M. (2021). mTORC1 stimulates cell growth through SAM synthesis and m(6)A mRNA-dependent control of protein synthesis. Mol. Cell 81, 2076–2093 e2079. 10.1016/j.molcel.2021.03.009 PMC8141029 33756106

[B135] Vu L. P. Pickering B. F. Cheng Y. Zaccara S. Nguyen D. Minuesa G. (2017). The N(6)-methyladenosine (m(6)A)-forming enzyme METTL3 controls myeloid differentiation of normal hematopoietic and leukemia cells. Nat. Med. 23, 1369–1376. 10.1038/nm.4416 28920958 PMC5677536

[B136] Wang J. Li Y. Wang P. Han G. Zhang T. Chang J. (2020). Leukemogenic chromatin alterations promote AML leukemia stem cells *via* a KDM4C-ALKBH5-AXL signaling axis. Cell Stem Cell 27, 81–97 e88. 10.1016/j.stem.2020.04.001 32402251

[B137] Weng H. Huang H. Wu H. Qin X. Zhao B. S. Dong L. (2018). METTL14 inhibits hematopoietic stem/progenitor differentiation and promotes leukemogenesis via mRNA m(6)A modification. Cell Stem Cell 22, 191–205 e199. 10.1016/j.stem.2017.11.016 29290617 PMC5860916

[B138] Weng Y. Xue J. Niu N. (2024). SETD2 in cancer: functions, molecular mechanisms, and therapeutic regimens. Cancer Biol. Med. 21, 725–730. 10.20892/j.issn.2095-3941.2024.0201 39302028 PMC11414219

[B139] Xiao M. Yang H. Xu W. Ma S. Lin H. Zhu H. (2012). Inhibition of alpha-KG-dependent histone and DNA demethylases by fumarate and succinate that are accumulated in mutations of FH and SDH tumor suppressors. Genes Dev. 26, 1326–1338. 10.1101/gad.191056.112 22677546 PMC3387660

[B140] Xu L. Li Q. Wang Y. Wang L. Guo Y. Yang R. (2021). m(6)A methyltransferase METTL3 promotes oral squamous cell carcinoma progression through enhancement of IGF2BP2-mediated SLC7A11 mRNA stability. Am. J. Cancer Res. 11, 5282–5298. PMC8640804 34873461

[B141] Xu M. Xu C. Wang R. Tang Q. Zhou Q. Wu W. (2025). Treating human cancer by targeting EZH2. Genes Dis. 12, 101313. 10.1016/j.gendis.2024.101313 40028035 PMC11870178

[B142] Yang C. Hu Y. Zhou B. Bao Y. Li Z. Gong C. (2020). The role of m(6)A modification in physiology and disease. Cell Death Dis. 11, 960. 10.1038/s41419-020-03143-z 33162550 PMC7649148

[B143] Yang J. Xu J. Wang W. Zhang B. Yu X. Shi S. (2023a). Epigenetic regulation in the tumor microenvironment: molecular mechanisms and therapeutic targets. Signal Transduct. Target Ther. 8, 210. 10.1038/s41392-023-01480-x PMC10203321 37217462

[B144] Yang X. Xu L. Yang L. (2023b). Recent advances in EZH2-based dual inhibitors in the treatment of cancers. Eur. J. Med. Chem. 256, 115461. 10.1016/j.ejmech.2023.115461 37156182

[B145] Yang Y. Jianxu Z. Hao L. Nini Q. Ge L. Jingjie Y. (2025). EF24 targets METTL3 to reprogram m6A methylation and induce ferroptosis: an epitranscriptomic mechanism with therapeutical potential for glioma. Cell Commun. Signal 24, 32. 10.1186/s12964-025-02583-4 PMC12817625 41388311

[B146] Yang Y. Zhang X. Venkadakrishnan V. B. Zou H. Zheng X. Guo S. (2026). Targeting wnt/Beta-Catenin and circadian regulator restores PRC2/EZH2-controlled chromatin bivalency and suppresses cell state diversity. J. Clin. Invest. 136, e200260. 10.1172/jci200260 PMC13132380 41842971

[B147] Yu H. Y. Yang L. Liu Y. C. Yu A. J. (2023). Sulforaphene suppressed cell proliferation and promoted apoptosis of COV362 cells in endometrioid ovarian cancer. PeerJ 11, e16308. 10.7717/peerj.16308 38025760 PMC10668859

[B148] Zeng C. Huang W. Li Y. Weng H. (2020). Roles of METTL3 in cancer: mechanisms and therapeutic targeting. J. Hematol. Oncol. 13, 117. 10.1186/s13045-020-00951-w 32854717 PMC7457244

[B149] Zhang C. Samanta D. Lu H. Bullen J. W. Zhang H. Chen I. (2016a). Hypoxia induces the breast cancer stem cell phenotype by HIF-Dependent and ALKBH5-mediated m(6)A-demethylation of NANOG mRNA. Proc. Natl. Acad. Sci. U. S. A. 113, E2047–E2056. 10.1073/pnas.1602883113 27001847 PMC4833258

[B150] Zhang R. L. Peng L. X. Yang J. P. Zheng L. S. Xie P. Wang M. Y. (2016b). IL-8 suppresses E-cadherin expression in nasopharyngeal carcinoma cells by enhancing E-cadherin promoter DNA methylation. Int. J. Oncol. 48, 207–214. 10.3892/ijo.2015.3226 26530812

[B151] Zhang S. M. Cai W. L. Liu X. Thakral D. Luo J. Chan L. H. (2021). KDM5B promotes immune evasion by recruiting SETDB1 to silence retroelements. Nature 598, 682–687. 10.1038/s41586-021-03994-2 34671158 PMC8555464

[B152] Zhang F. Liu H. Duan M. Wang G. Zhang Z. Wang Y. (2022). Crosstalk among m(6)A RNA methylation, hypoxia and metabolic reprogramming in TME: from immunosuppressive microenvironment to clinical application. J. Hematol. Oncol. 15, 84. 10.1186/s13045-022-01304-5 35794625 PMC9258089

[B153] Zhang D. Deng Y. Kukanja P. Agirre E. Bartosovic M. Dong M. (2023a). Spatial epigenome-transcriptome co-profiling of Mammalian tissues. Nature 616, 113–122. 10.1038/s41586-023-05795-1 PMC10076218 36922587

[B154] Zhang M. Lu A. Wang H. Yang J. (2023b). Quercetin downregulates the expression of IL15 in cancer cells through DNA methylation. Eur. Rev. Med. Pharmacol. Sci. 27, 2580–2590. 10.26355/eurrev_202303_31795 37013776

[B155] Zhang Q. C. Qian Y. M. Ren Y. H. Chen M. M. Cao L. M. Zheng S. J. (2024a). Phenethyl isothiocyanate inhibits metastasis potential of non-small cell lung cancer cells through FTO mediated TLE1 m(6)A modification. Acta Pharmacol. Sin. 45, 619–632. 10.1038/s41401-023-01178-4 PMC10834501 37848553

[B156] Zhang S. Zhou L. Yang J. Lu J. Tao L. Feng Y. (2024b). Rhein exerts anti-multidrug resistance in acute myeloid leukemia via targeting FTO to inhibit AKT/mTOR. Anticancer Drugs 35, 597–605. 10.1097/cad.0000000000001608 38728095

[B157] Zhao S. Allis C. D. Wang G. G. (2021). The language of chromatin modification in human cancers. Nat. Rev. Cancer 21, 413–430. 10.1038/s41568-021-00357-x 34002060 PMC10507815

[B158] Zheng G. Dahl J. A. Niu Y. Fedorcsak P. Huang C. M. Li C. J. (2013). ALKBH5 is a Mammalian RNA demethylase that impacts RNA metabolism and mouse fertility. Mol. Cell 49, 18–29. 10.1016/j.molcel.2012.10.015 23177736 PMC3646334

[B159] Zhra M. Holail J. H. Mohammad K. S. (2025). Epigenetic modulation and bone metastasis: evolving therapeutic strategies. Pharm. (Basel) 18, 1140. 10.3390/ph18081140 PMC12389651 40872531

[B160] Zhu J. Dai G. Chen T. Zhou Y. Zang Y. Xu L. (2024). Ailanthone suppresses cell proliferation of renal cell carcinoma partially via inhibition of EZH2. Discov. Oncol. 15, 464. 10.1007/s12672-024-01347-9 39298003 PMC11413286

